# New azaphilones from *Aspergillus neoglaber*

**DOI:** 10.1186/s13568-020-01078-4

**Published:** 2020-08-17

**Authors:** Thomas Isbrandt, Jens C. Frisvad, Anja Madsen, Thomas O. Larsen

**Affiliations:** Department of Biotechnology and Biomedicine, Technical University of Denmark, Kongens Lyngby, Denmark

**Keywords:** Azaphilones, Aspergillus neoglaber, Pigments, Structural elucidation, Tandem mass spectrometry

## Abstract

Three new azaphilones, sassafrin E (**1**), sassafrin F (**2**), and sassafrinamine A (**3**), were isolated from the filamentous fungus *Aspergillus neoglaber*. The structures of the compounds were determined by nuclear magnetic resonance spectroscopy, and were found to be novel analogues of two already known compound classes; sassafrins and berkchaetoazaphilones. Sassafrin E and F were both oxygen containing, while sassafrinamine A additionally contained a nitrogen atom, originating from an aminoethanol moiety, as well as extensive conjugation resulting in an intense purple colour of the pure compound. The structure of sassafrin E was further confirmed using deuterium exchange experiments coupled with high-resolution tandem mass spectrometry.

## Key points

We present three new azaphilone pigments isolated from the fungus *Aspergillus neoglaber*. Two pigments (Sassafrin E and Sassafrin F) were yellow and the third (sassafrinamine A) purple. The structure of Sassafrin E was supported by tandem MS experiments of the deuterium labelled molecule.

## Introduction

The use of natural or naturally derived pigments for the food and cosmetics industry has in recent years been of increasing interest, and filamentous fungi have long been known to produce various colourful secondary metabolites. Several of such compounds exist within the class of azaphilones, such as atrorosins, a group of red pigments that we recently characterised (Isbrandt et al. 2020). Azaphilones are common in the three large genera *Aspergillus*, *Penicillium* and *Talaromyces* (Osmanova et al. 2010; Samson et al. 2011; Romsdahl and Wang 2019). In *Aspergillus*, azaphilones have been found in the subgenera *Circumdati* and *Nidulantes*, but they are less common in subgenus *Fumigati* (Samson et al. 2007; Frisvad and Larsen 2016). *Aspergillus fumigatus* in subgenus *Fumigati* section *Fumigati* has been reported to produce *epi*-pinophilin B and other azaphilones (Zhang et al. 2019), and *Aspergillus clavatus* in subgenus *Fumigati* section *Clavati* has been reported to produce small azaphilones, such as felinone A and aspergillusone B (Wang et al. 2015).

One strategy for identifying new compounds is by looking into the secondary metabolites of underexplored species. The secondary metabolites of the filamentous fungus *Aspergillus neoglaber* (= *Neosartorya glabra*) from section *Fumigati* have not been well described, and only little work has been done to gain a better understanding of which compounds are produced by this species (Samson et al. 2007; Jayasuriya et al. 2009; Liu et al. 2015; Frisvad and Larsen 2016; May Zin et al. 2016). A few of the reported secondary metabolites include the bioactive glabramycins (Jayasuriya et al. 2009; Li 2015) and satoryglabrins (Liu et al. 2015), as well as various diketopiperazines and tetracyclopeptides (May Zin et al. 2016). When cultivated on solid media, we found *Aspergillus neoglaber* to color the surrounding media red, suggesting production of one or several red-pigmented compounds, none of which have been characterized as yet. This discovery prompted us to further investigate the nature of these predicted new metabolites, and thereby shed some light on this underexplored species.

## Materials and methods

### Solvents and instrumentation

All solvents were acquired from Sigma-Aldrich (St. Louis, Missouri, USA), ultra-pure water was made with a Milli-Q system (Millipore, Burlington, Massachusetts, USA).

Ultra-high Performance Liquid Chromatography-High Resolution Tandem Mass Spectrometry (UHPLC-HRMS/MS) was performed on an Agilent Infinity 1290 UHPLC system (Agilent Technologies, Santa Clara, CA, USA) equipped with a diode array detector. Separation was obtained on an Agilent Poroshell 120 phenyl-hexyl column (2.1 × 250 mm, 2.7 μm) with a linear gradient consisting of water (A) and acetonitrile (B) both buffered with 20 mM formic acid, starting at 10% B and increased to 100% in 15 min where it was held for 2 min, returned to 10% in 0.1 min and remaining for 3 min (0.35 mL/min, 60 °C). MS detection was performed in positive detection mode on an Agilent 6545 QTOF MS equipped with Agilent Dual Jet Stream electrospray ion source with a drying gas temperature of 250 °C, gas flow of 8 L/min, sheath gas temperature of 300 °C and flow of 12 L/min. Capillary voltage was set to 4000 V and nozzle voltage to 500 V. Mass spectra were recorded at 10, 20 and 40 eV as centroid data for *m*/*z* 85–1700 in MS mode and *m*/*z* 30–1700 in MS/MS mode, with an acquisition rate of 10 spectra/s. Lock mass solution in 70:30 methanol:water was infused in the second sprayer using an extra LC pump at a flow of 15 μL/min using a 1:100 splitter. The solution contained 1 μM tributylamine (Sigma-Aldrich) and 10 μM Hexakis (2,2,3,3-tetrafluoropropoxy)phosphazene (Apollo Scientific Ltd., Cheshire, UK) as lock masses. The [M + H]^+^ ions (*m*/*z* 186.2216 and 922.0098 respectively) of both compounds were used.

1D and 2D NMR spectra were recorded on a Bruker Avance 600 MHz or Bruker Avance 800 MHz spectrometer (Bruker, Billerica, MA, USA). NMR spectra were acquired using standard pulse sequences. The solvent used was CD_3_OD, and residual MeOH-d3 was used as references with signals at δ_H_ = 3.31 ppm and δ_C_ = 49.0 ppm. Data processing and analysis was done using TopSpin 3.5pl7 (Bruker). *J*-couplings are reported in hertz (Hz) and chemical shifts in ppm (δ).

Deuterium exchange of **1** was achieved by leaving the compound in CD_3_OD for 5 days at 5 °C.

### Strain and purification

The strain used for this study was *Aspergillus neoglaber* IBT 3020, obtained from the DTU strain collection. For large scale extractions, the fungus was grown in six 2L conical flasks each with 500 mL of yeast extract sucrose (YES) medium containing only 10% of the normal amount of agar.

Biomass and media was separated by decantation, and extraction was done twice on the biomass, using ethyl acetate (EtOAc) acidified with 1% formic acid. Initial fractionation of the extract was done on an Isolera One (Biotage) flash system using a diol column eluted stepwise with dichloromethane (DCM), DCM:EtOAc (1:1), EtOAc, EtOAc:MeOH (1:1), and MeOH. Final isolation of the pure compounds was done using a semi-preparative Waters 600 Controller with a 996 photodiode array detector (Waters, Milford, MA, USA) equipped with a Luna II C18 column (250 × 10 mm, 5 μm, Phenomenex), using a H_2_O/acetonitrile gradient with 50 ppm TFA.

## Results

**Fig. 1:**
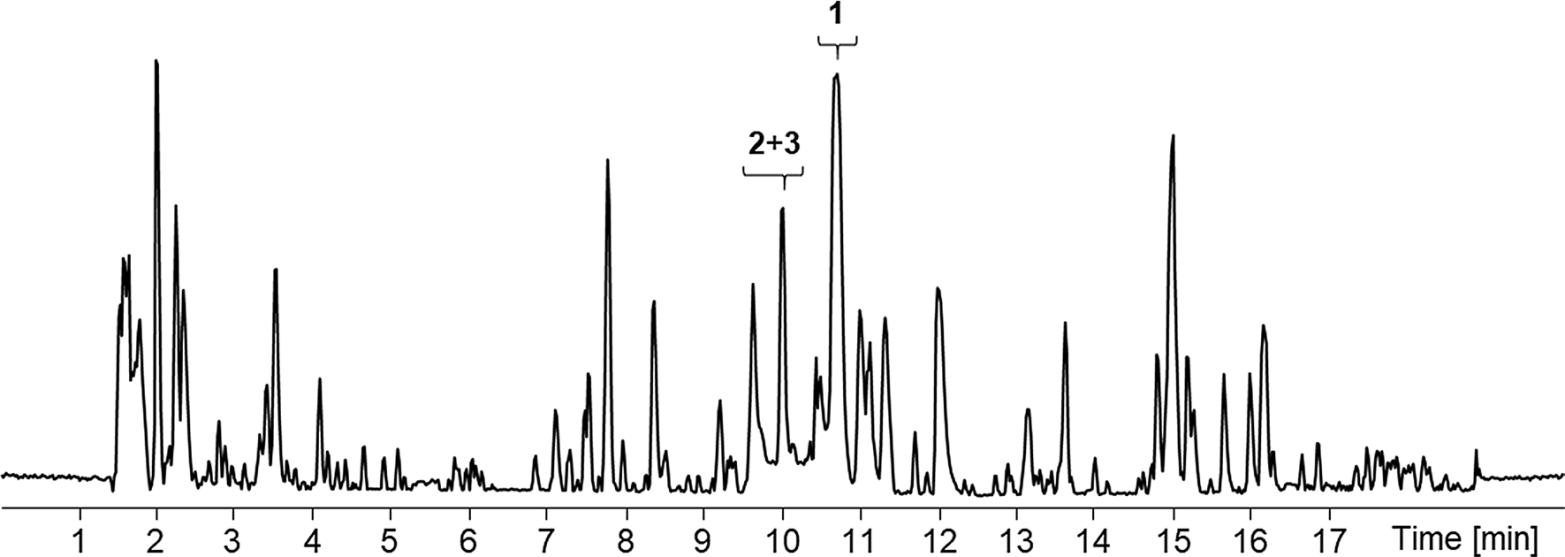
Base peak chromatogram (LC-DAD-HRMS, ESI +) of the ethyl acetate extract from *A. neoglaber* grown on YES medium

Analysis by ultra-high performance liquid chromatography coupled to diode array detection and high resolution tandem mass spectrometry (UHPLC-DAD-HRMS/MS) of the ethyl acetate extract from the filamentous fungus *Aspergillus neoglaber* IBT 3020 = CBS 111.55, which is the ex-type culture of the species (Fig. 1), identified the major secondary metabolite as a yellow compound (**1**) absorbing at 345 nm with an m/z of 453.2277 Da ([M + H]^+^) and molecular formula C_27_H_32_O_6_. A second, also yellow compound (**2**) absorbing at 360 nm and with a mass and molecular formula corresponding to the addition of two protons to **1** was tentatively identified as a likely analogue (m/z 455.2428, [M + H]^+^, C_27_H_34_O_6_). In addition to the two yellow compounds, a third, red–purple compound (**3**) absorbing at 545 nm with a m/z of 494.2541, corresponding to a molecular formula of C_29_H_36_NO_6_, and found to be responsible for the red colour of the extract was also identified. UV–VIS spectra acquired during HPLC analysis for each of the three compounds can be found in Additional file 1: Fig. S1.

In order to purify the three compounds, *A. neoglaber* IBT 3020 was cultivated on 6 × 500 mL semi-liquid YES media. The biomass was extracted with ethyl acetate, and purification was done using normal phase flash chromatography followed by semi-preparative RP-HPLC. One- and two-dimensional NMR experiments were used in order to elucidate the structures of the compounds.

**Fig. 2:**
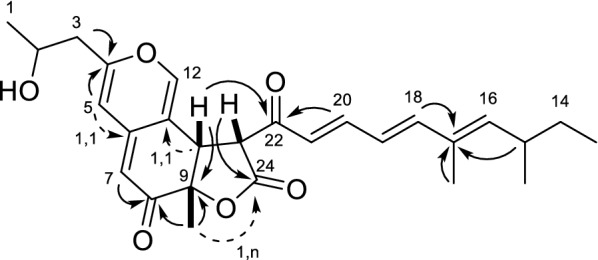
Structure and key HMBC (plain arrows) and ADEQUATE (dashed arrows) correlations for sassafrin E (**1**)

In compound **1**, a total of 31 protons could be identified from the ^1^H-spectrum, suggesting one exchangeable proton. In combination with multiplicity edited HSQC (edHSQC), 12 CH-groups, two CH_2_-groups, and five CH_3_-groups could be identified. Eight of the CH-groups had carbon shifts matching alkenes, and one CH-group was identified to be attached to a hydroxyl group, accounting for the 32^nd^ proton not observed in the ^1^H-spectrum. Both CH_2_-groups appeared as diastereotopic. ^3^*J* H–H couplings obtained from DQF-COSY, identified four spin systems consisting of **H1** to **H3**, **H13** to **H16** and **C15-CH**_**3**_, **H18** to **H21**, and **H10** and **H23**, as well as five singlets. Correlations in the DQF-COSY, were all confirmed by H2BC. HMBC correlations linked **H15**, **C17–CH**_**3**_ and **H18** to **C17**, and **H10** and **H20** to **C22**. Additionally, **H1**, **H2** and **H3**, along with **H5** was linked to **C4**. Ambiguity in the HMBC around **C6** and **C11** meant that additional, more specific experiments were needed, and 1,n- and 1,1-ADEQUATE (Reif et al. 1996) experiments were used to connect **H5** and **H7** to **C6**, **H10** to **C11**, as well as **C9-CH**_**3**_ to **C24** (Fig. 2). NOESY correlations around the lactone could assign relative stereochemistry to the methyl group **C9-CH**_**3**_ and the two protons **H10** and **H23**. In summary, compound **1** turned out to be a novel azaphilone, with high structural similarity to groups of compounds such as sassafrins (Quang et al. 2005) and berkchaetoazaphilones (Stierle et al. 2015), and has been named sassafrin E.

The NMR data for compounds **2** and **3**, was highly similar to that of **1**, with only few variations. Compound **2** was determined to only differ from **1**, by having the ketone at **C22** being reduced to a hydroxyl group and has been named sassafrin F.

**Fig. 3:**
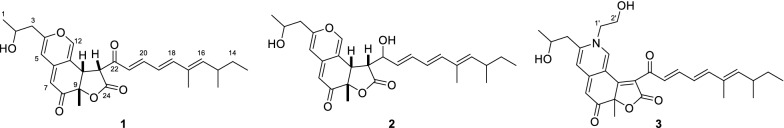
Numbered structures of sassafrin E (**1**), sassafrin F (**2**), and sassafrinamine A (**3**). Relative configuration is shown for **C9**, **C10** and **C23**

Compared to compound **1,** compound **3** (m/z 494.2541, [M + H]^+^, C_29_H_36_NO_6_) included three additional hydrogen atoms, two more carbon atoms, as well as a nitrogen. From the edHSQC spectrum, the additional carbon atoms were determined to be two CH_2_-groups (**C1′** and **C2′**). HMBC correlations from **H12** to **C1′** determined the two-carbon moiety to be an N-linked aminoethanol, generating an isoquinoline ring as shown in Fig. 3. Furthermore, no protons could be linked to carbons **C10** and **C23**, and it is therefore assumed that these are connected via a double bond. Similarly, no correlations to **C6** were observed. The UV–VIS spectrum for **3** was quite unique, with slight absorption all the way from 270 nm to 580 nm, with maximum at 545 nm, and the extensive conjugation is in agreement with the violet/purple colour of the pure compound. Compound **3** has been named sassarinamine A,^1^ based on the incorporation of nitrogen, a key feature of azaphilones. Structures of compounds **1**, **2**, and **3** are shown in Fig. 3 and chemical shifts are listed in Table 1. Recorded NMR spectra for each compounds can be found in Additional file 1.

**Tables 1:** ^1^H and ^13^C NMR shifts for compounds **1**, **2**, and **3**

#	Sassafrin E (**1**)		Sassafrin F (**2**)		Sassafrinamine A (**3**)	
	δ_H_	δ_C_	δ_H_	δ_C_	δ_H_	δ_C_
1	1.22	23.4	1.23	22.1	1.33	23.6
2	4.06	66.2	4.01	65.3	4.15	67.9
3	2.52/2.59	43.7	2.55	42.3	2.95/3.01	41.5
4	–	162.9	–	161.3	–	154.7
5	6.34	109.9	6.34	108.5	7.08	122.4
6	–	148.2	–	147.3	–	n/a
7	5.41	105.7	5.38	104	6.86	99.1
8	–	194.1	–	193.3	–	196
9	–	84.3	–	82.7	–	86.5
9-CH3	1.55	23.5	1.55	21.5	1.69	30.1
10	4.03	44.6	3.63	42.1	–	n/a
11	–	116.4	–	114.8	–	119.9
12	7.53	149.8	7.42	150	8.35	144.4
13	0.86	12.3	0.87	10.9	0.87	12
14	1.30/1.42	31.2	1.29/1.42	30.1	1.31/1.43	31
15	2.48	36.1	2.45	34.3	2.48	35.6
15-CH3	0.99	20.6	0.99	19.6	0.99	20.5
16	5.60	147.4	5.32	139.7	5.51	144.9
17	–	134.4	–	138.1	–	134.2
17-CH3	1.82	12.6	1.78	11.4	1.85	12.4
18	6.77	150.4	6.3	138.1	6.68	146.9
19	6.40	125.7	6.15	125	6.45	126.4
20	7.41	148.4	6.4	131.1	7.36	142.4
21	6.46	128.2	5.65	132.4	7.55	127.9
22	–	192.4	4.83	68.1	–	185.8
23	4.41	55.7	3.01	48.7	–	n/a
24	–	171.3	–	174.4	–	173.6
1′					4.32/4.51	57.7
2′					3.89	61.3

In addition to the NMR experiments, we were able to further confirm the structure of sassafrin E by exchanging **H23** with deuterium, and using the isotope labelled fragments in tandem MS experiments to generate the MS/MS spectra found in Additional file 1: Fig. S2 and S3. In this way, three destinct fragmentation pathways for the molecule could be suggested as depicted in Additional file 1: Fig. S4.

## Discussion

During the initial stage of this study, **1** was suspected to be the acetylcholineesterase inhibitor arisugacin C (Otoguro et al. 1997, 2000) or an analogue hereof. However, dereplication by comparison of retention time, absorption spectra, and fragmentation pattern (Additional file 1: Fig. S5) with an extract from *Penicillium echinulatum*, a known producer of arisugacin C, quickly clarified that the compound in *A. neoglaber* was not an arisugacin, but rather belonging to an entirely different class of compounds.

**Fig. 4:**
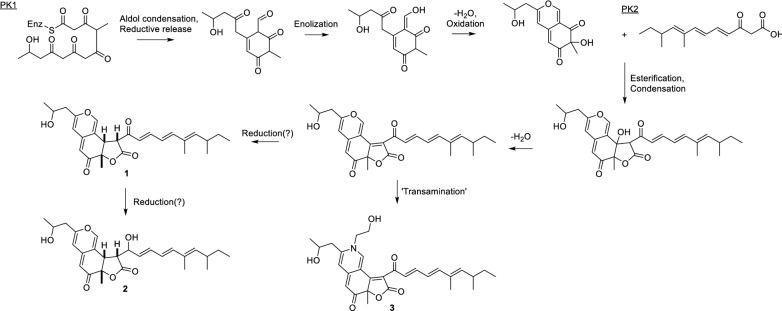
Proposed biosynthetic pathway, for compounds **1**, **2** and **3**

The isolated compounds were found to be members of the compound class azaphilones, a diverse group of compounds, such as the ones obtained from various *Monascus* species, i.e. the so-called *Monascus* pigments (Gao et al. 2013), including the newly described subclass atrorosins of which we recently reported nineteen new analogues (Isbrandt et al. 2020). The exact biosynthetic pathway of these compounds have not been fully elucidated, although some work has been done in order to propose a possible route (Hajjaj et al. 1999; Somoza et al. 2012; Liu et al. 2014; Woo et al. 2015; Chen et al. 2017; Tolborg et al. 2017). We therefore expect that the compounds described in this study are biosynthesised in a similar fashion, as outlined in Fig. 4. However, in contrast to many azaphilone pigments, e.g. the *Monascus* pigments which are made from a hexaketide moiety and a 3-oxo-fatty acid, we propose sassafrin E and F, and sassafrinamine A to be constructed from two polyketides, due to the low level of reduction in the side chain (**C13** to **C24**). For Sassafrin F, we further expect the reduction of the ketone at **C22** to happen after construction of the compound backbone, as fungal PKSs are well known to be stringent with regard to the reduction pattern of their products, why it is unlikely that two different PKs would originate from the same biosynthetic PKS. This hypothesis is further strengthened when also considering the structures of the previously described sassafrins (Quang et al. 2005), all containing different levels of reduction in the side chain, as well as the berkchaetoazaphilones (Stierle et al. 2015) containing fully reduced side chains. The fusion of the lactone ring also differs from the linear fashion found in *Monascus* pigments and is more similar to compounds such as chaetoviridins (Winter et al. 2012).

As we have recently discovered for the compound class atrorosins (Isbrandt et al. 2020), the incorporation of nitrogen into the isochromene system can be done using various primary amine containing compounds (Tolborg et al. 2020). We speculate that the nitrogen, and additional carbons and oxygen in **3** originate from a decarboxylated serine moiety, i.e. ethanolamine which is abundant in cells as constituents in phospholipids in cell membranes (Wellner et al. 2013). Additionally, further investigation of the crude extract tentatively revealed an aminoethanol derivative of **2**, as well as the non-nitrogen containing precursor of **3** to be present in lower amounts. A common feature for both the compounds characterised in this study and the previously described atrorosins, is the observed CO loss during tandem MS experiments (see Additional file 1: Fig. S4).

## Supplementary information

**Additional file 1.** UV-, MS/MS and NMR spectra of sassafrin E, F and sassafrinamine A, as well as dereplication and  proposed MS/MS fragmentation pathway for sassafrin E.

## Data Availability

Not applicable.

## Footnotes

1 Low amounts of compound meant that signals for **C6**, **C10** and **C23** were not observed for **3**, and the tricyclic part of the structure was determined based on similarities with the remaining signals, compared to compound **1** and **2**.
