# Supplementary material for: New azaphilones from Aspergillus neoglaber
Source: AMB Express. 2020 Aug 17;10:145. doi: 10.1186/s13568-020-01078-4 (PMC7431503; doi:10.1186/s13568-020-01078-4)
Supplement: Supplementary file 1 — Additional file 1. UV-, MS/MS and NMR spectra of sassafrin E, F and sassafrinamine A, as well as dereplication and proposed MS/MS fragmentation pathway for sassafrin E. [file 13568_2020_1078_MOESM1_ESM.pdf]

AMB Express

New azaphilones from *Aspergillus neoglaber*

Thomas Isbrandt, Jens C. Frisvad, Anja Madsen, Thomas O. Larsen*

Department of Biotechnology and Biomedicine, Technical University of Denmark, Kongens Lyngby, Denmark

*Corresponding author (TOL). Phone: +45 45252632; email: tol@bio.dtu.dk

S1. UV-VIS spectra for sassafrin E, sassafrin F, and sassafrinamine A.

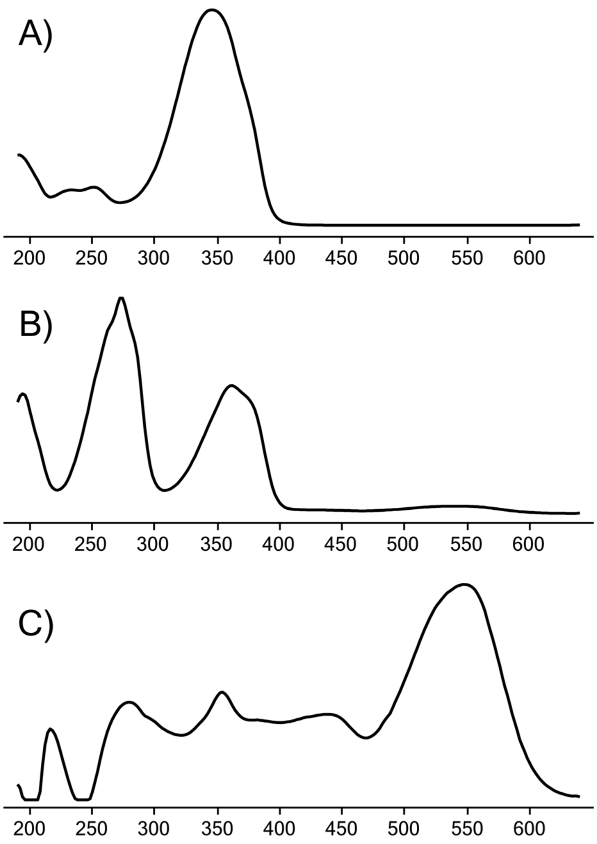

Figure S1. UV-VIS spectra for A) Sassafrin E, B) Sassafrin F, C) Sassafrinamine A. All spectra were recorded during UHPLC analysis in acetonitrile and H_2O, acidified with 20mM formic acid.

S2. NMR spectra for sasasfrin E (**1**), sassafrin F (**2**), and sassafrinamine A (**3**).

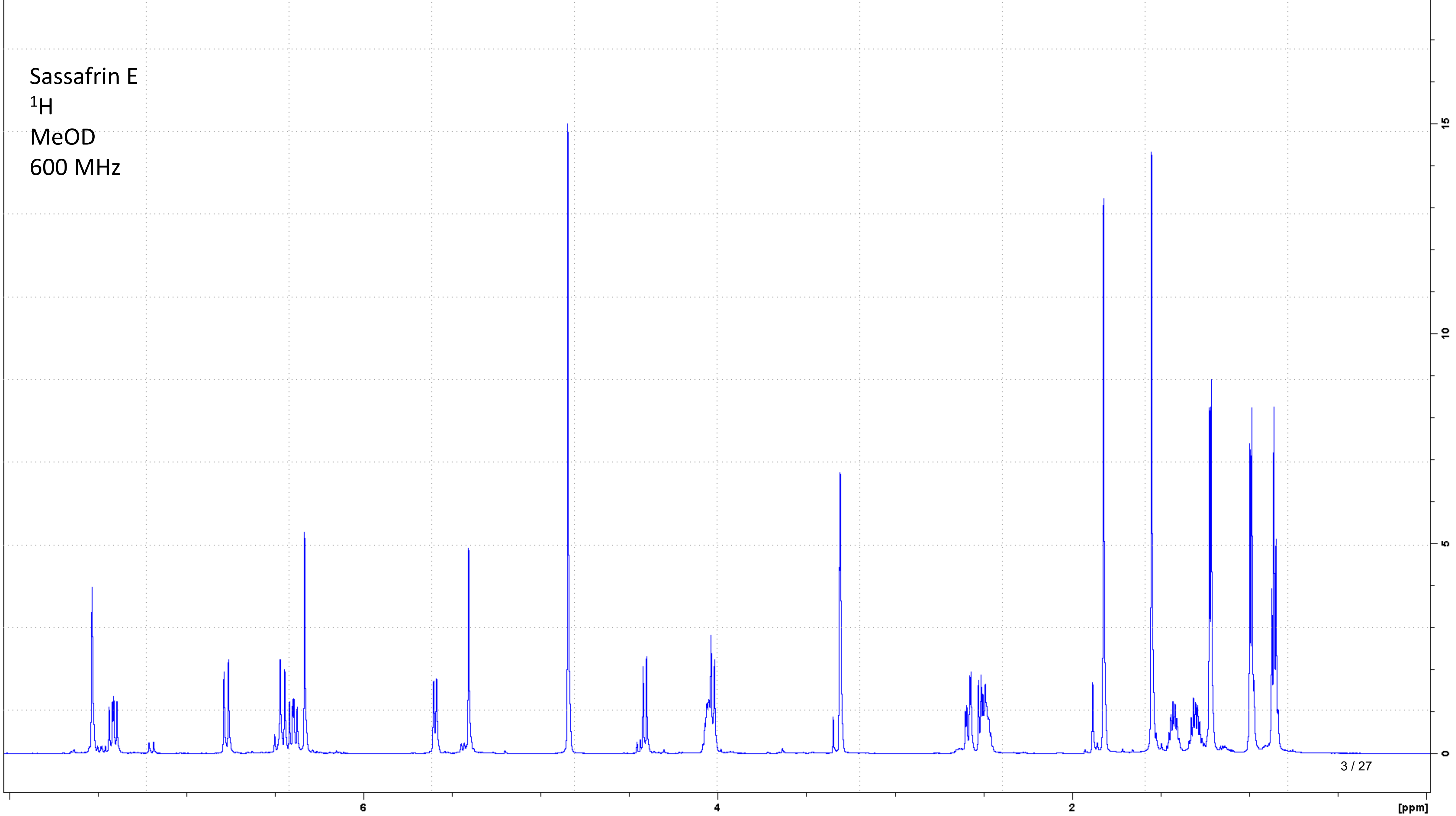

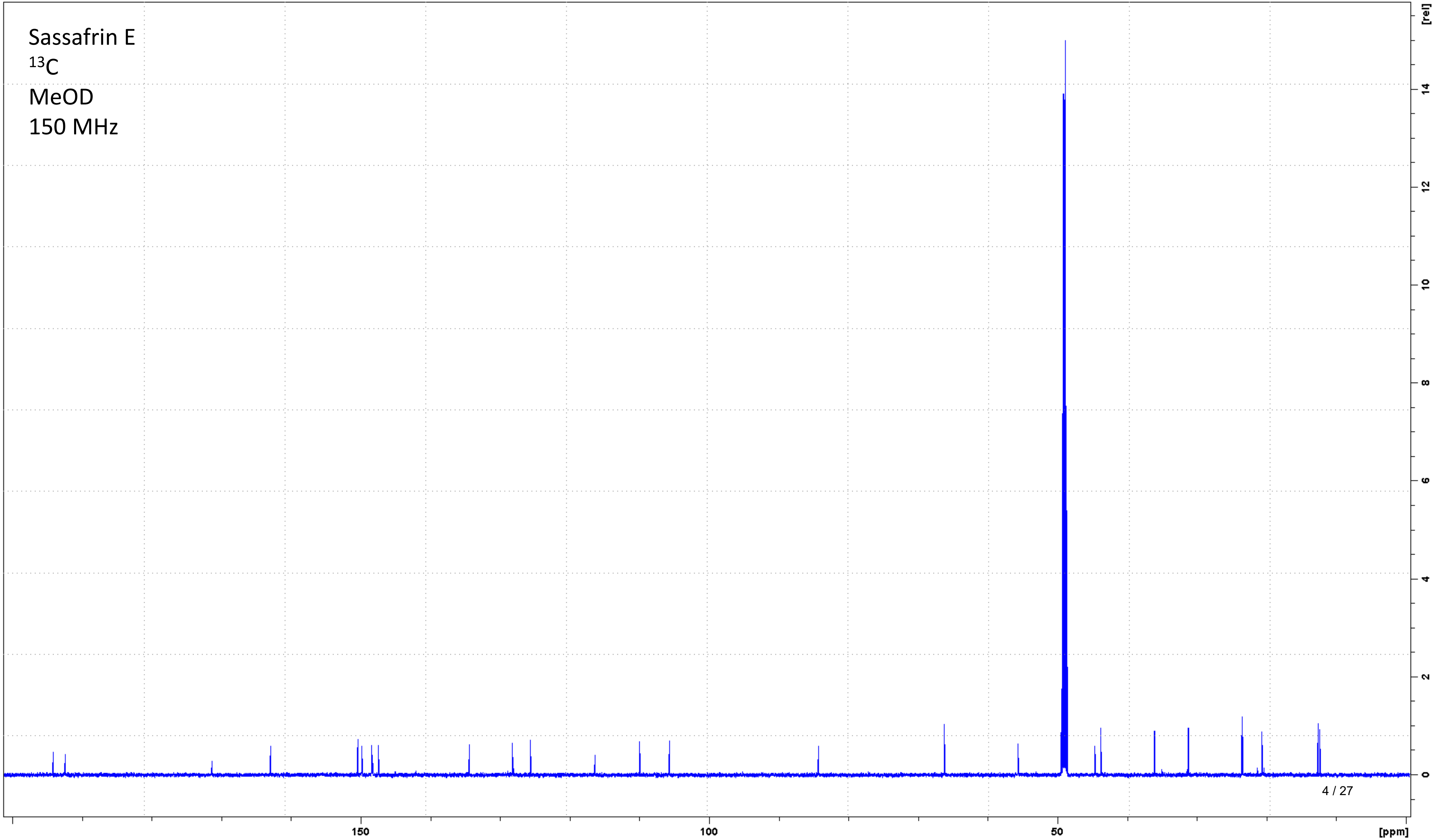
Sassafrin E
13C
MeOD
150 MHz
150
100
50
[ppm]
[rel]
0
2
4
6
8
10
12
14

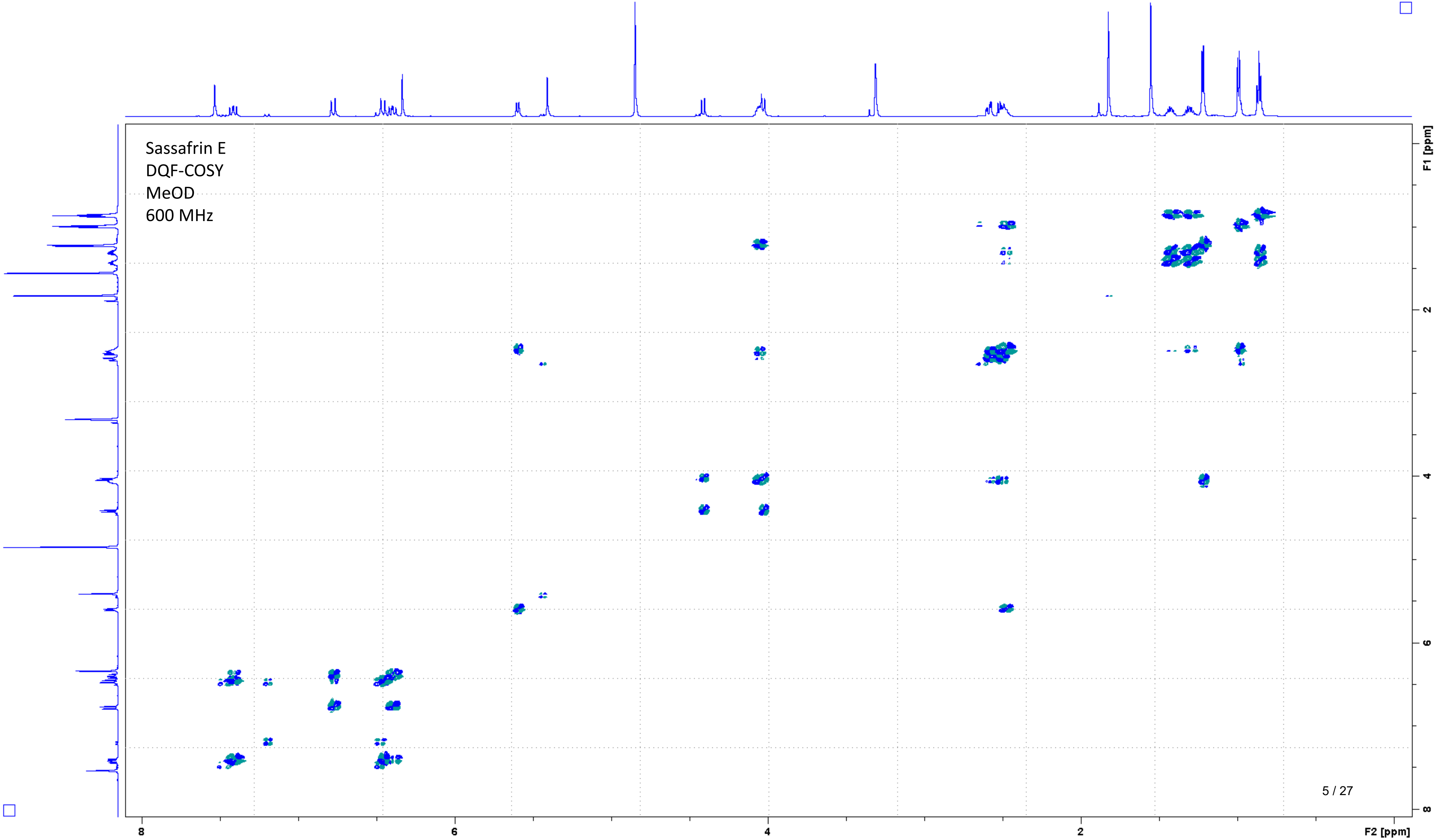

Sassafrin E
DQF-COSY
MeOD
600 MHz
F1 [ppm]
F2 [ppm]
8
6
4
2

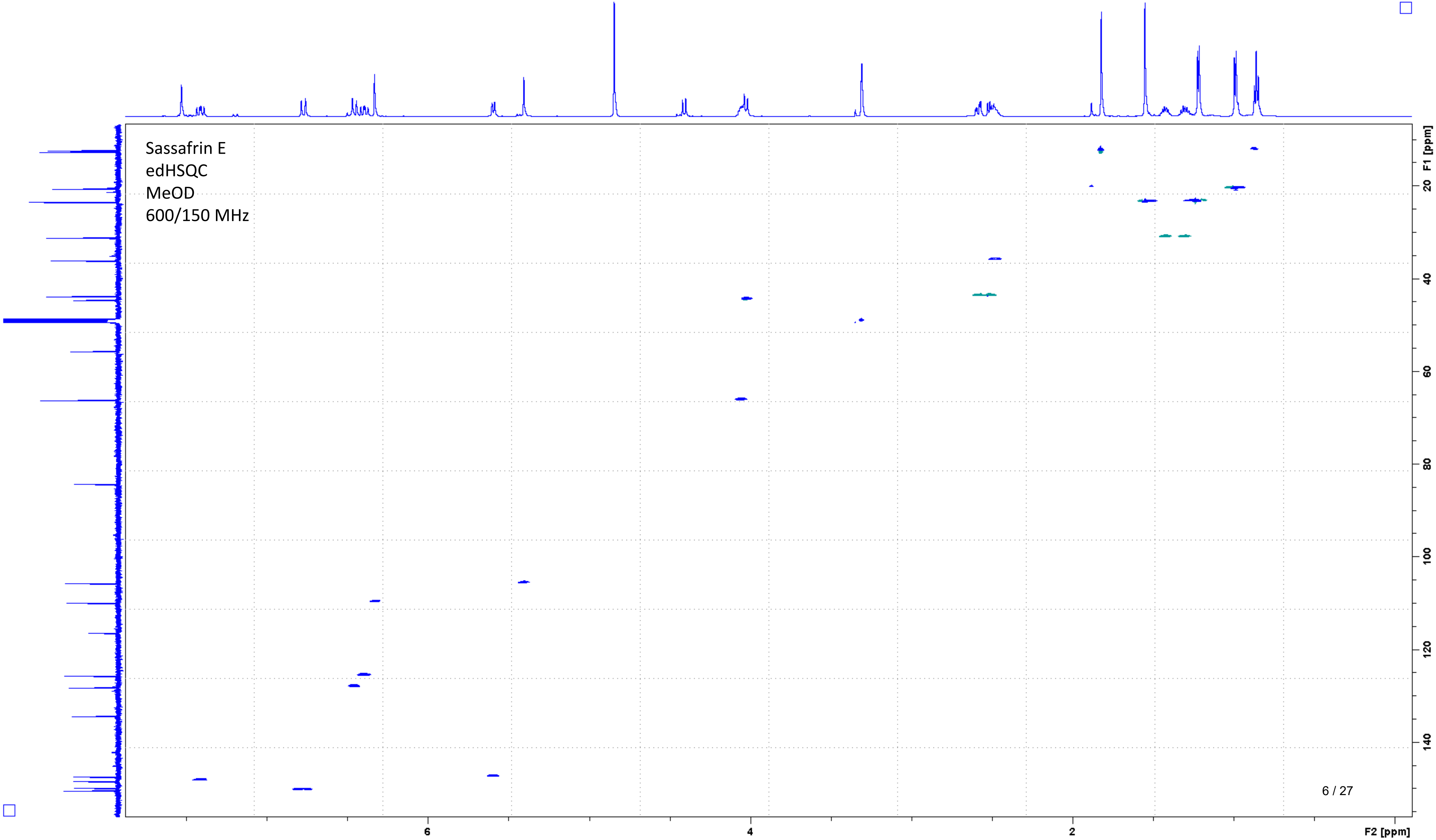
Sassafrin E
edHSQC
MeOD
600/150 MHz
F1 [ppm]
20
40
60
80
100
120
140
6
4
2
F2 [ppm]

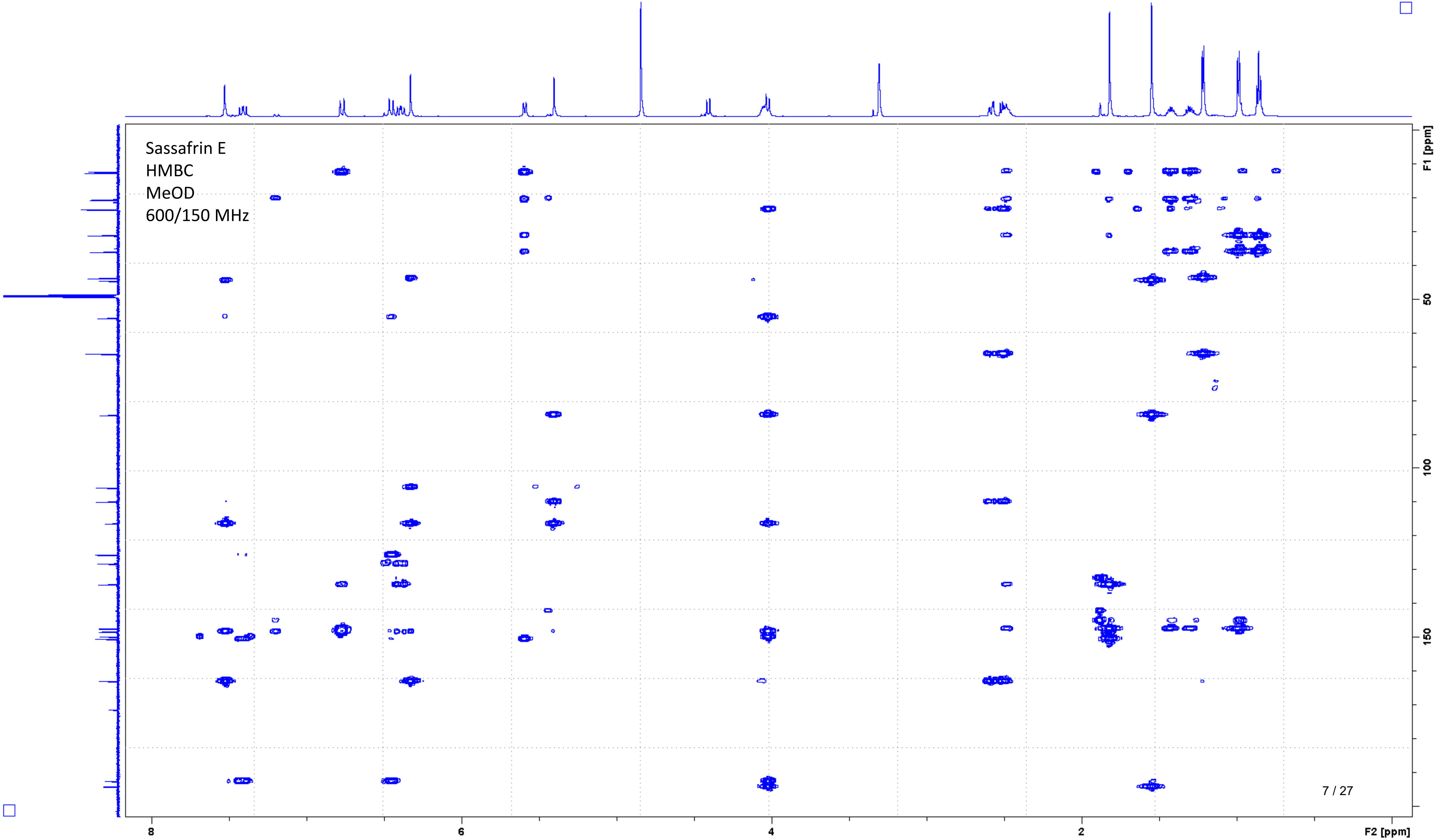
Sassafrin E
HMBC
MeOD
600/150 MHz
F1 [ppm]
50
100
150
8
6
4
2
F2 [ppm]

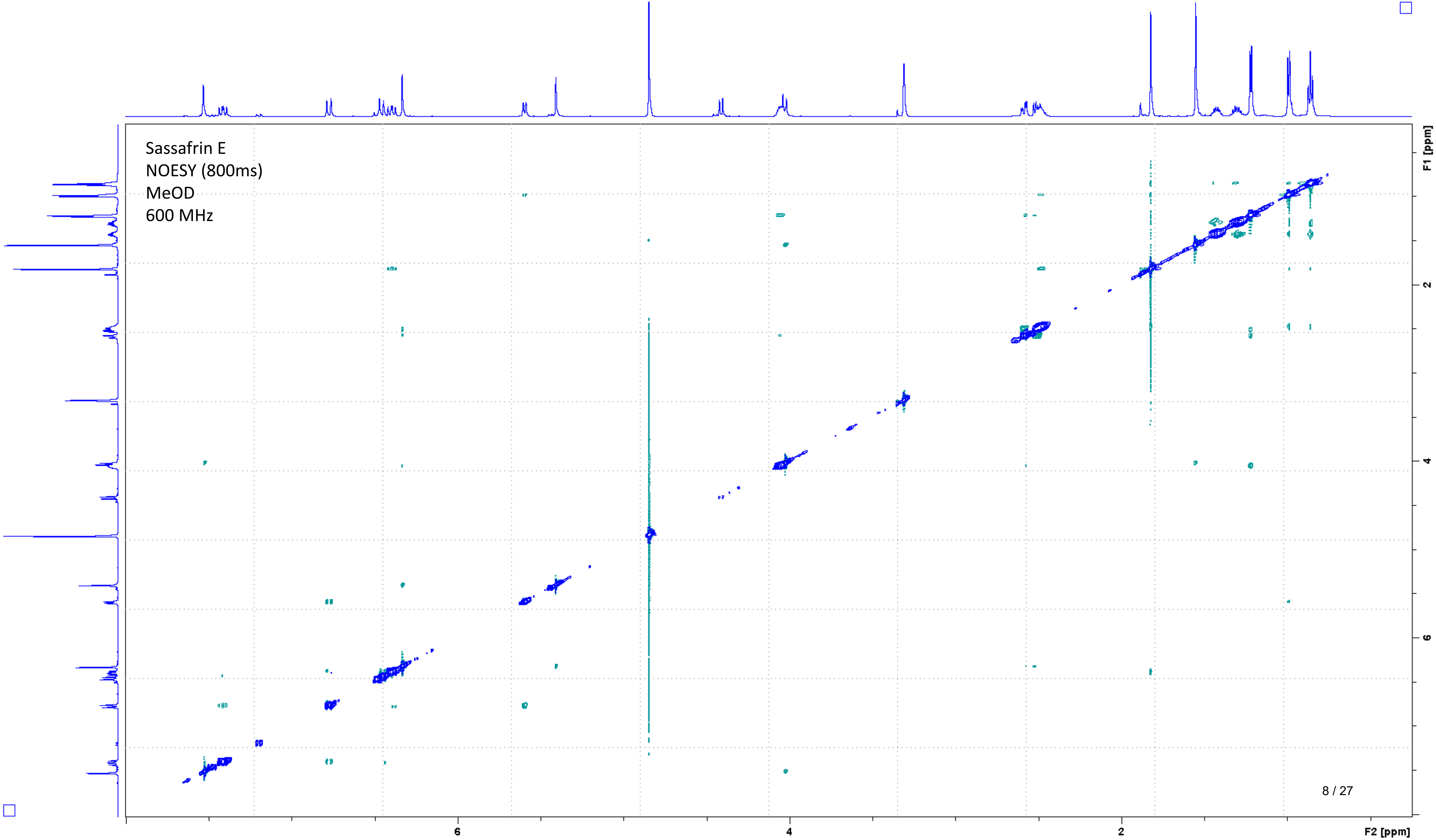
Sassafrin E
NOESY (800ms)
MeOD
600 MHz
F1 [ppm]
F2 [ppm]
2
4
6

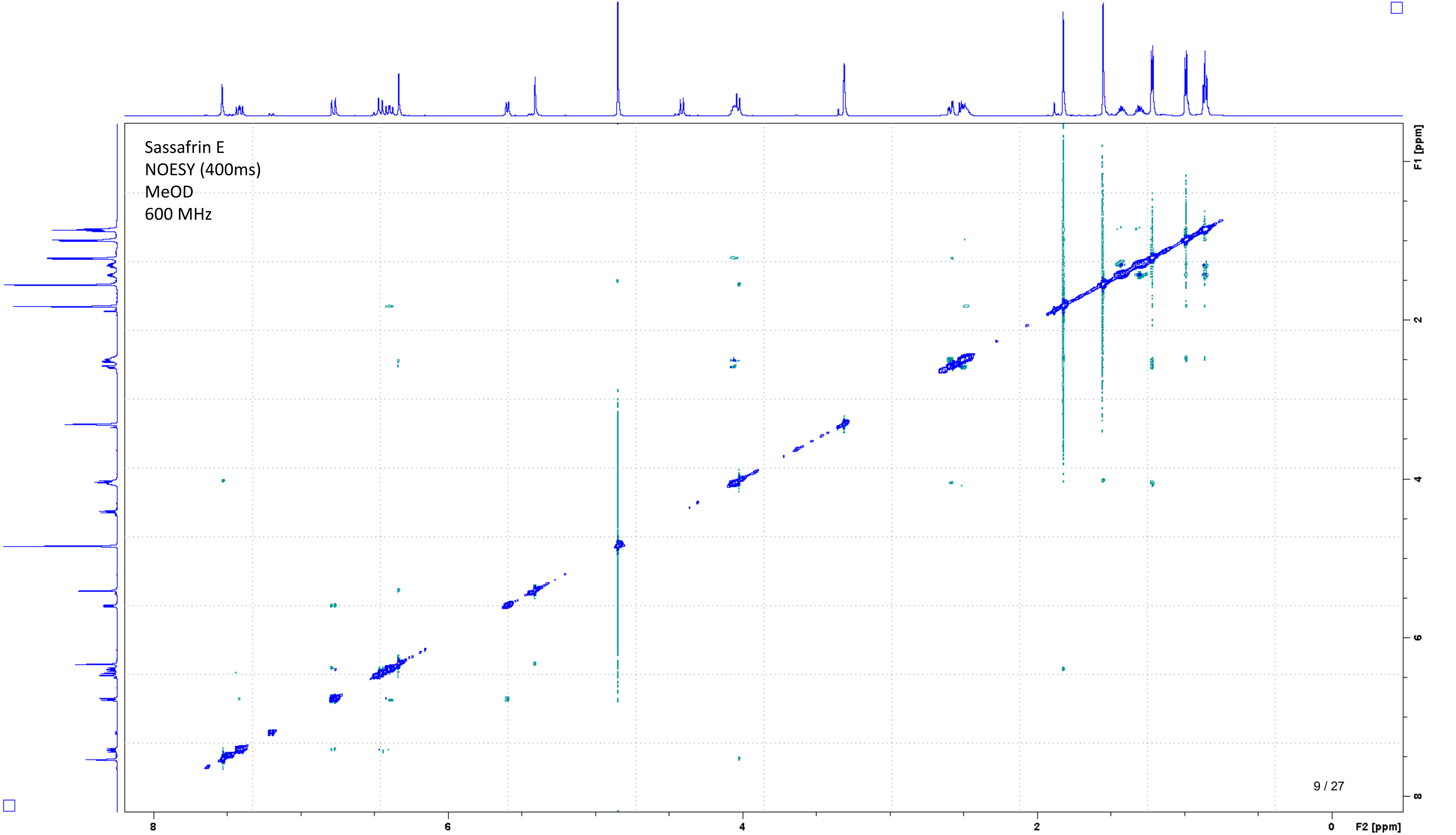

Sassafrin E
NOESY (400ms)
MeOD
600 MHz
F1 [ppm]
F2 [ppm]
8
6
4
2
0

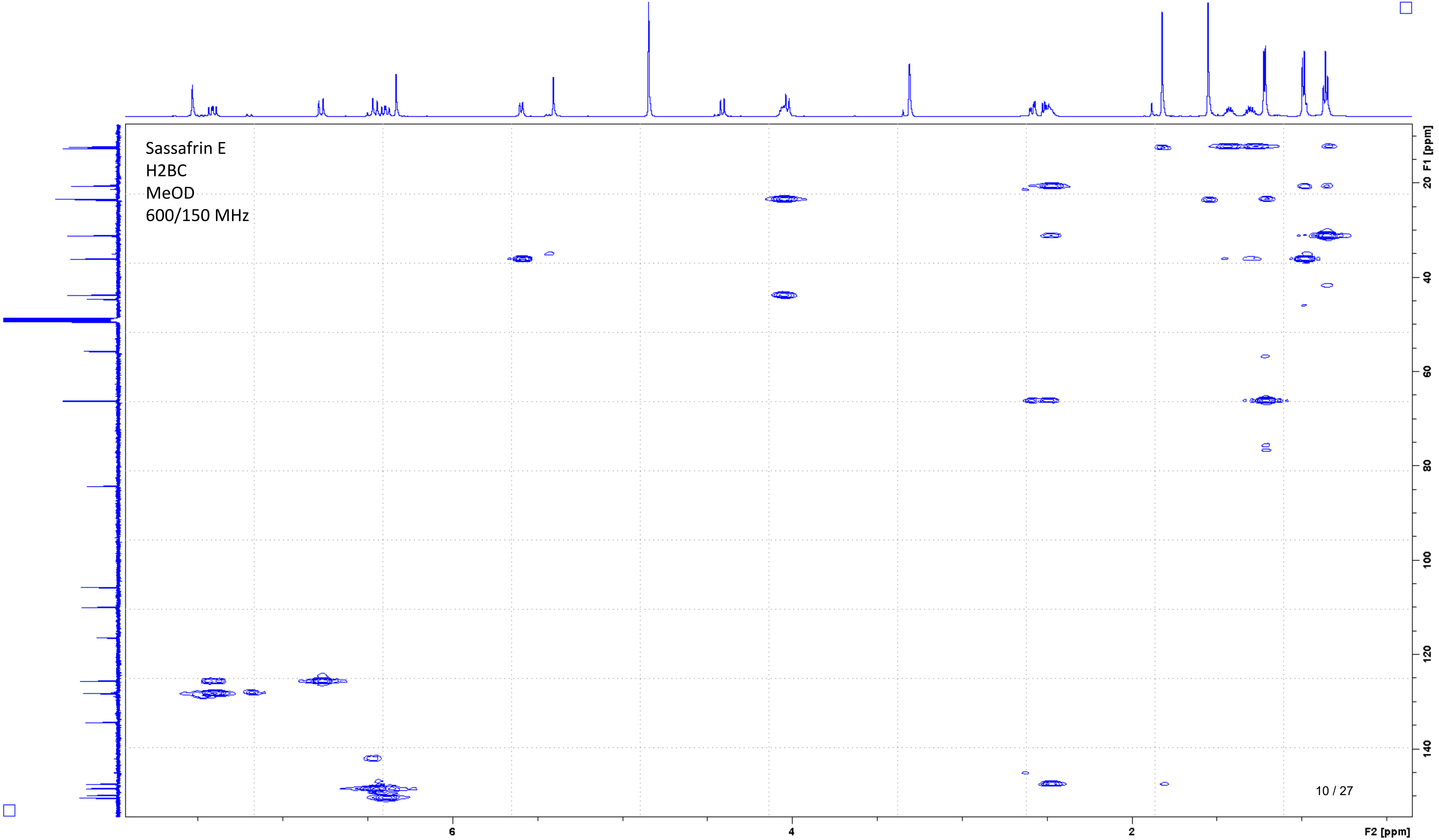
Sassafrin E
H2BC
MeOD
600/150 MHz
F2 [ppm]
F1 [ppm]

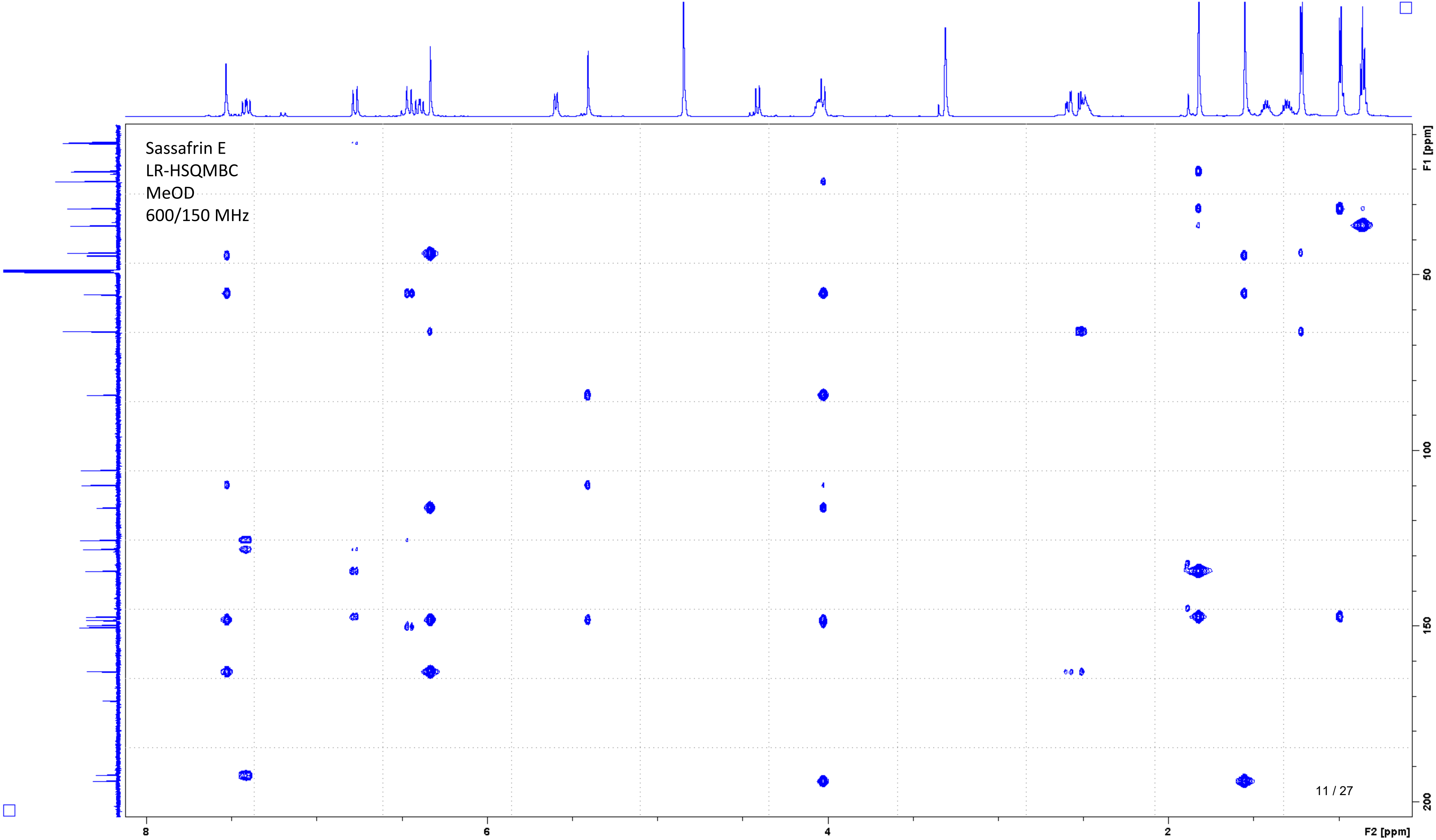
Sassafrin E
LR-HSQMBC
MeOD
600/150 MHz
F1 [ppm]
50
100
150
200
8
6
4
2
F2 [ppm]

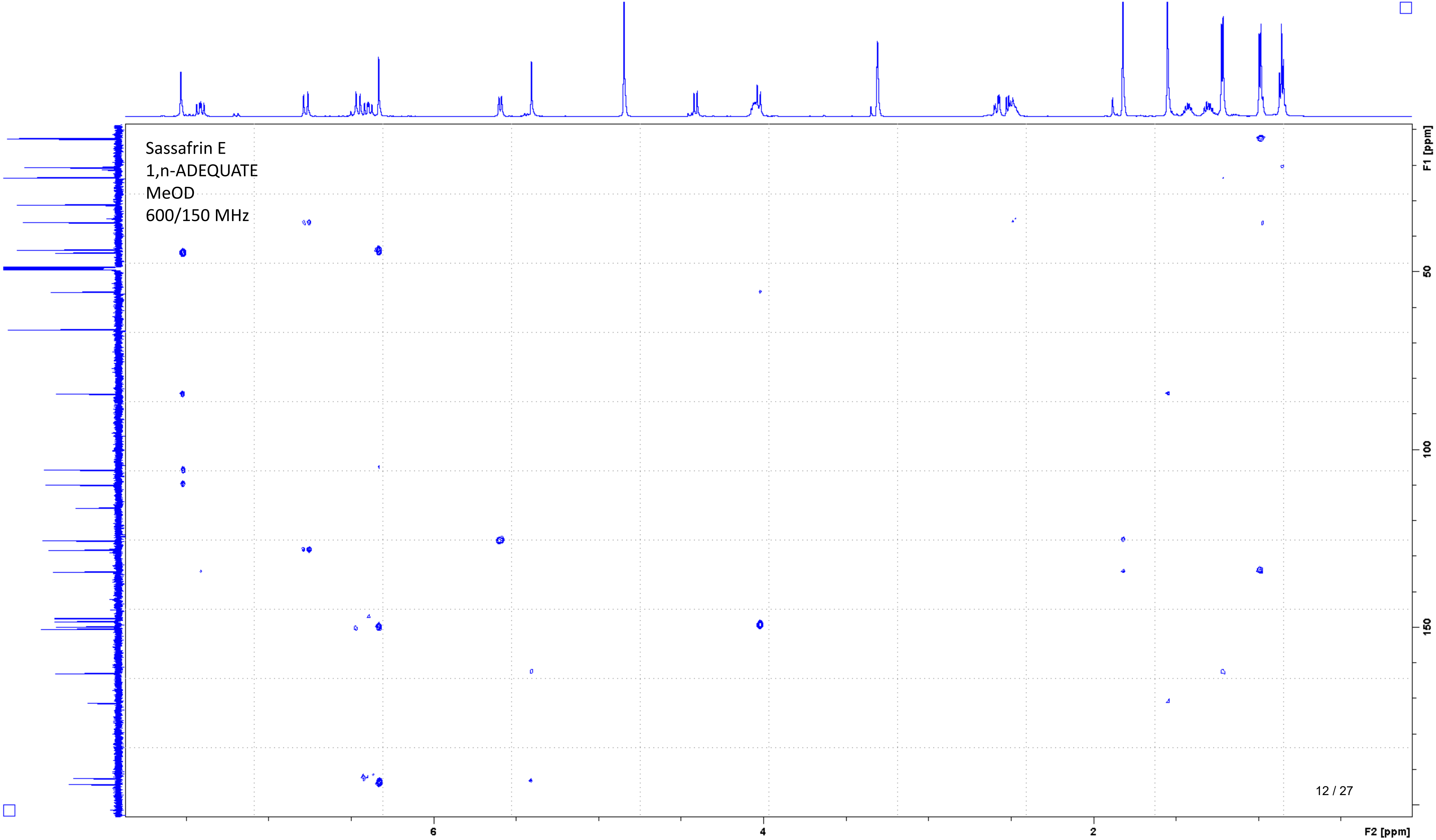
Sassafrin E
1,n-ADEQUATE
MeOD
600/150 MHz
F1 [ppm]
50
100
150
6
4
2
F2 [ppm]

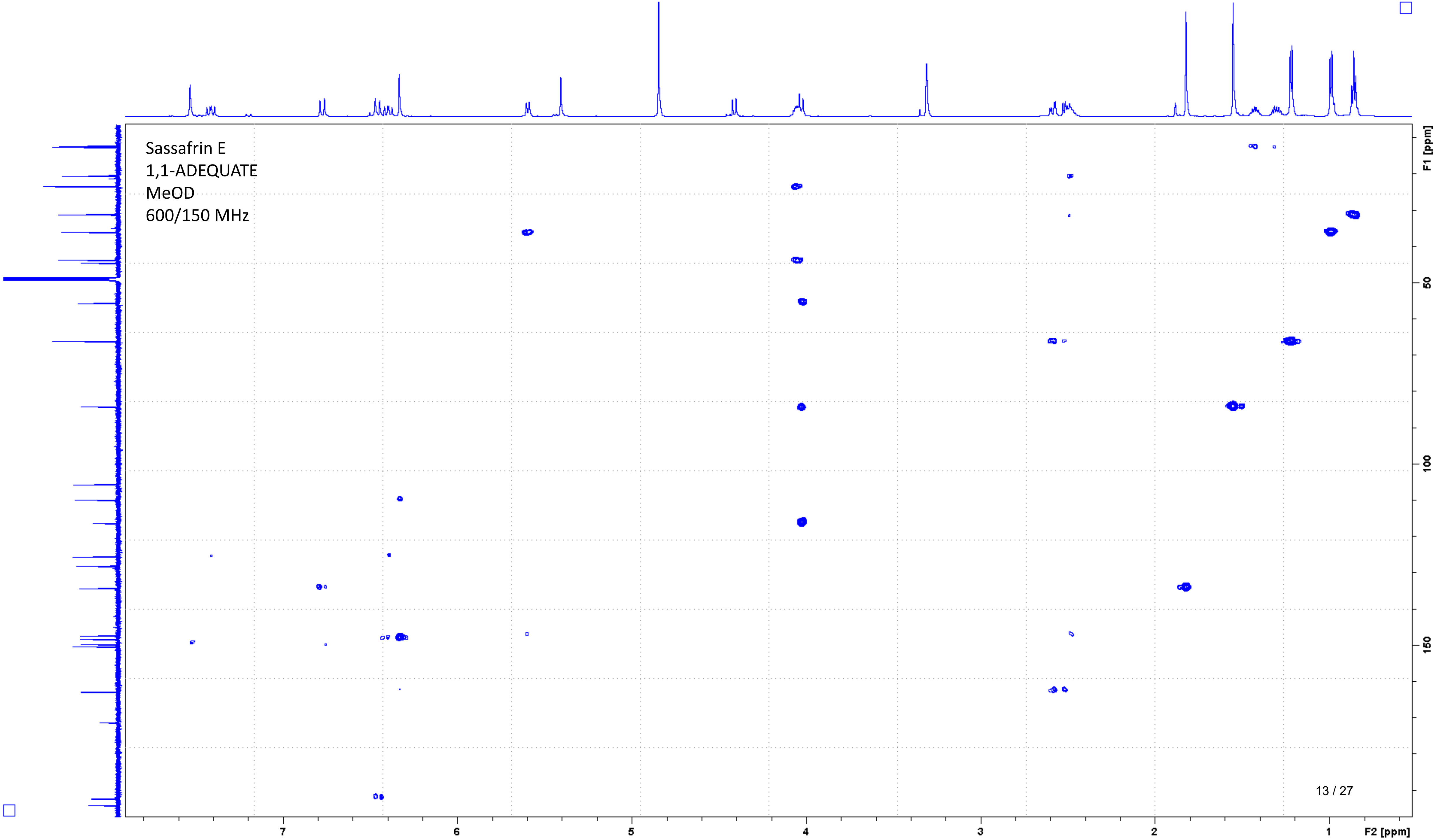

Sassafrin E
1,1-ADEQUATE
MeOD
600/150 MHz
F1 [ppm]
50
100
150
7
6
5
4
3
2
1
F2 [ppm]

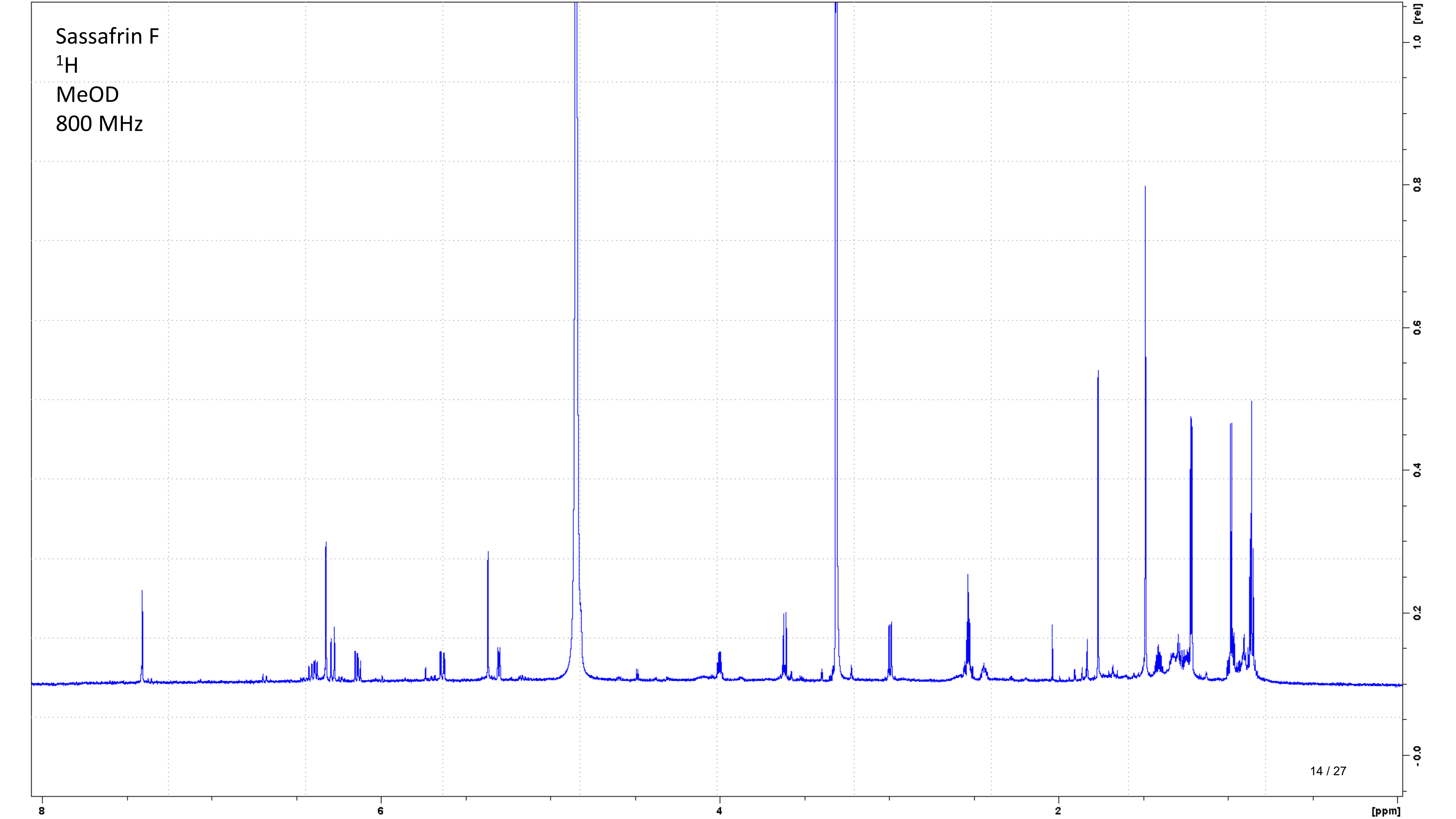
Sassafrin F
1H
MeOD
800 MHz
[rel]
[ppm]

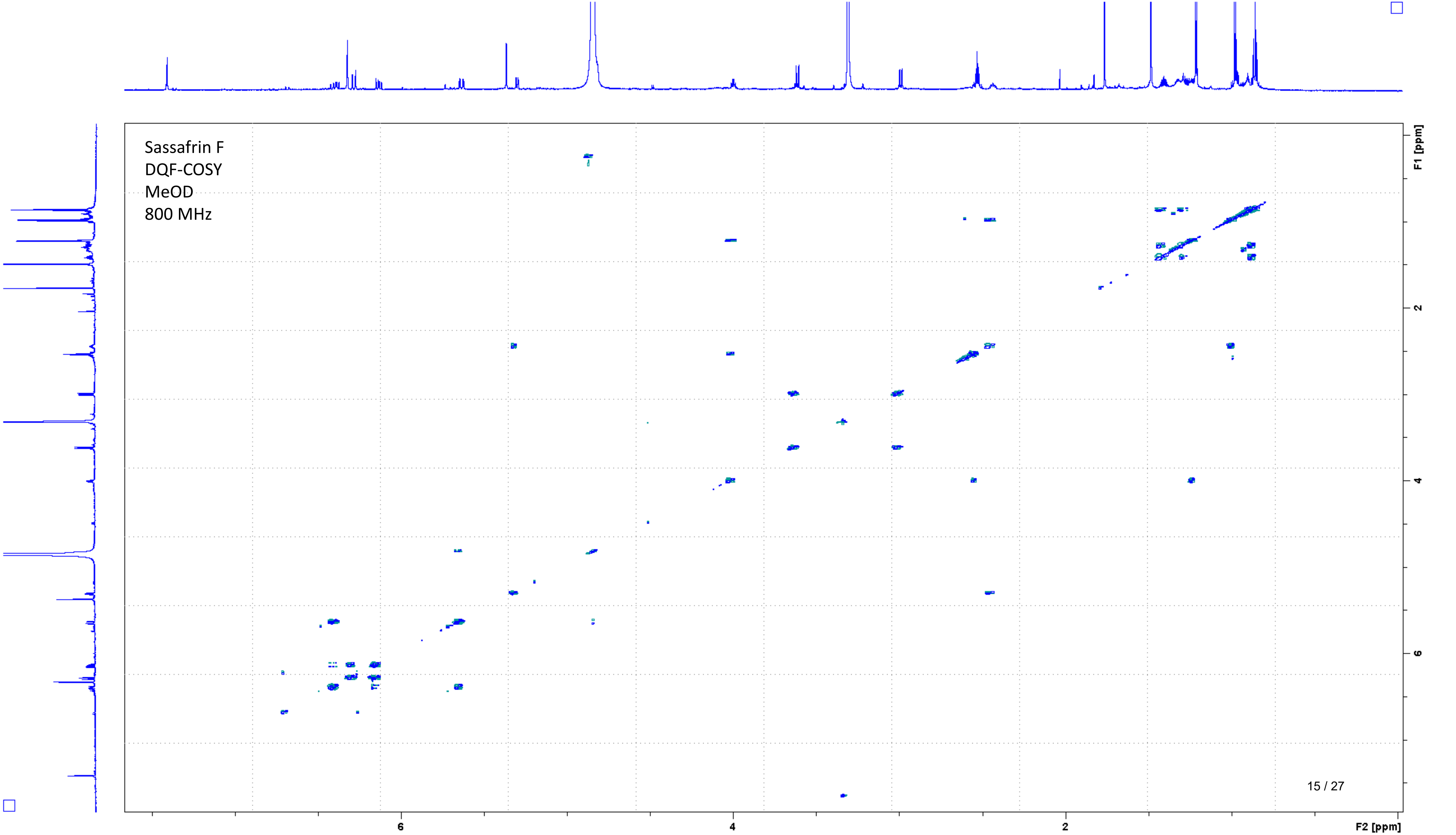
Sassafrin F
DQF-COSY
MeOD
800 MHz
F1 [ppm]
F2 [ppm]
6
4
2

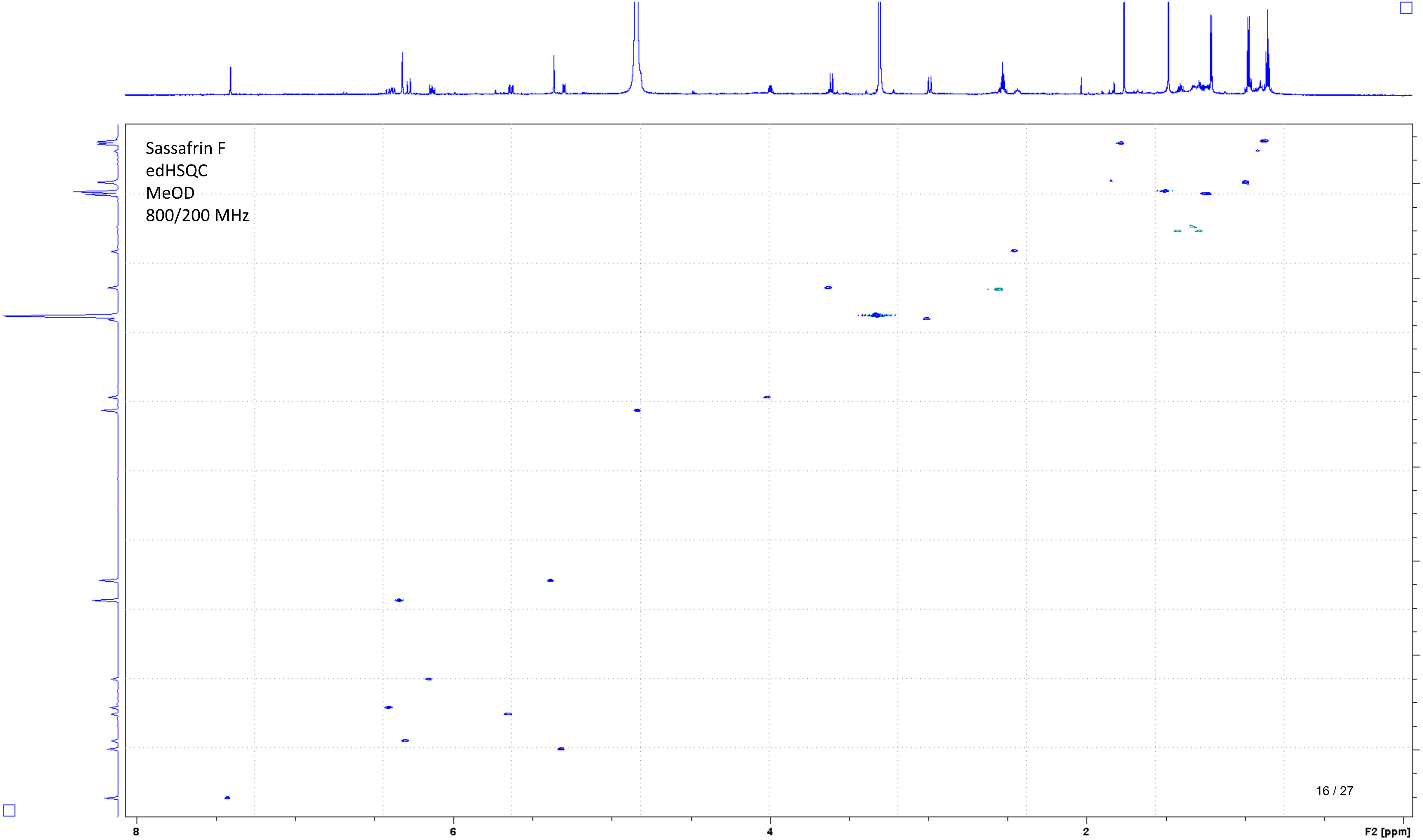

Sassafrin F
edHSQC
MeOD
800/200 MHz
F1 [ppm]
F2 [ppm]

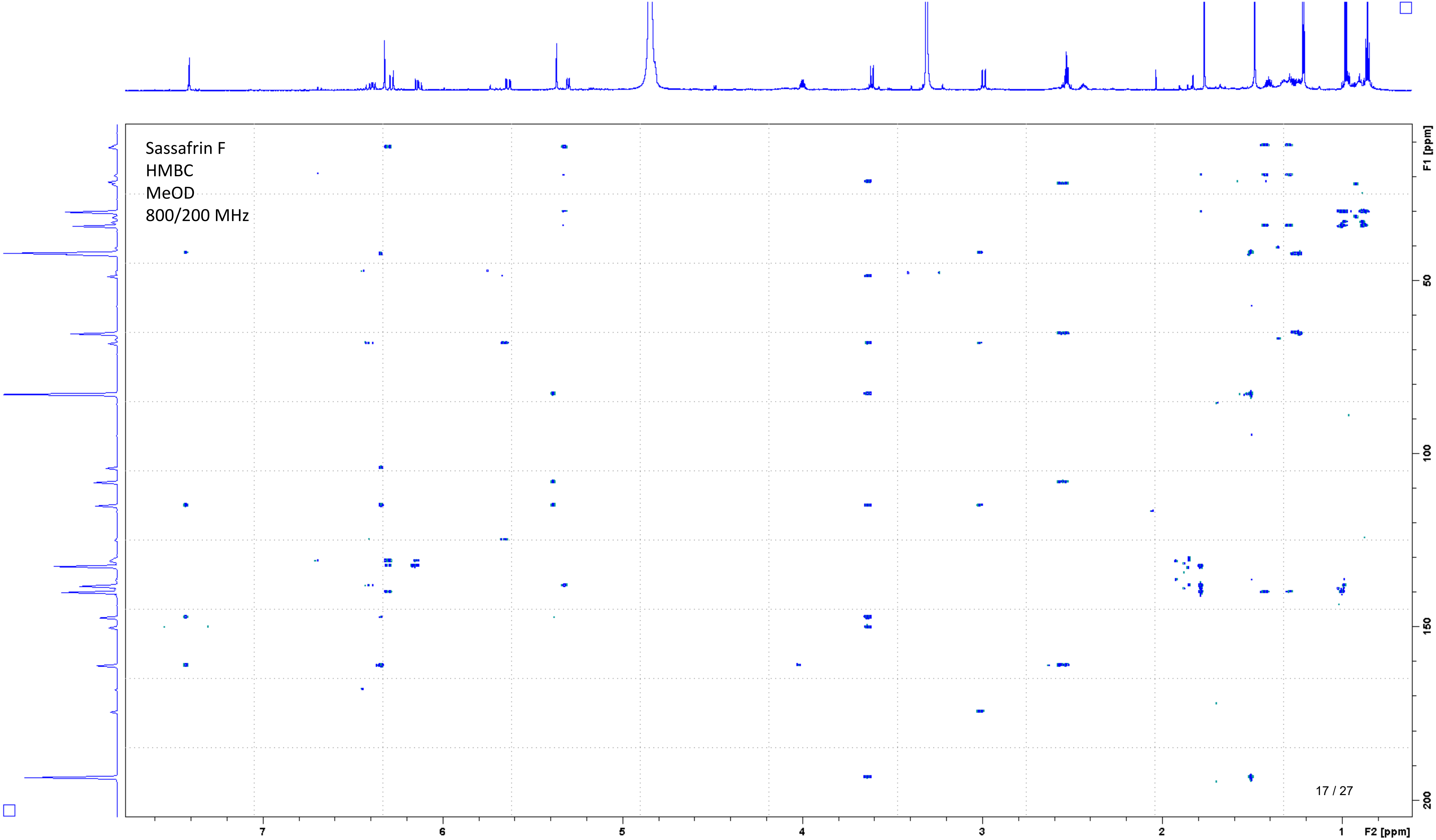
Sassafrin F
HMBC
MeOD
800/200 MHz
F1 [ppm]
F2 [ppm]
7
6
5
4
3
2
1
50
100
150
200

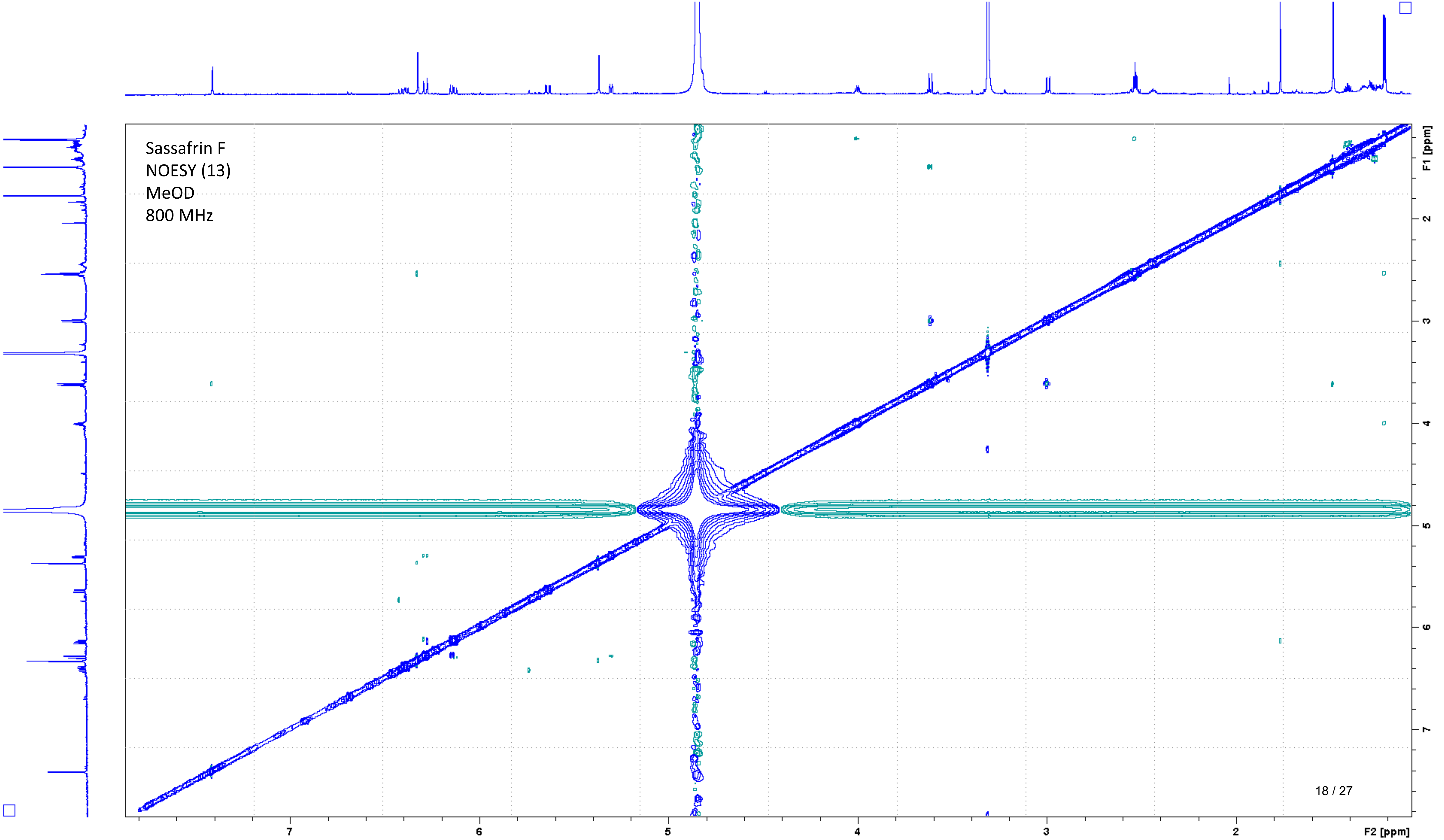

Sassafrin F
NOESY (13)
MeOD
800 MHz
F1 [ppm]
F2 [ppm]

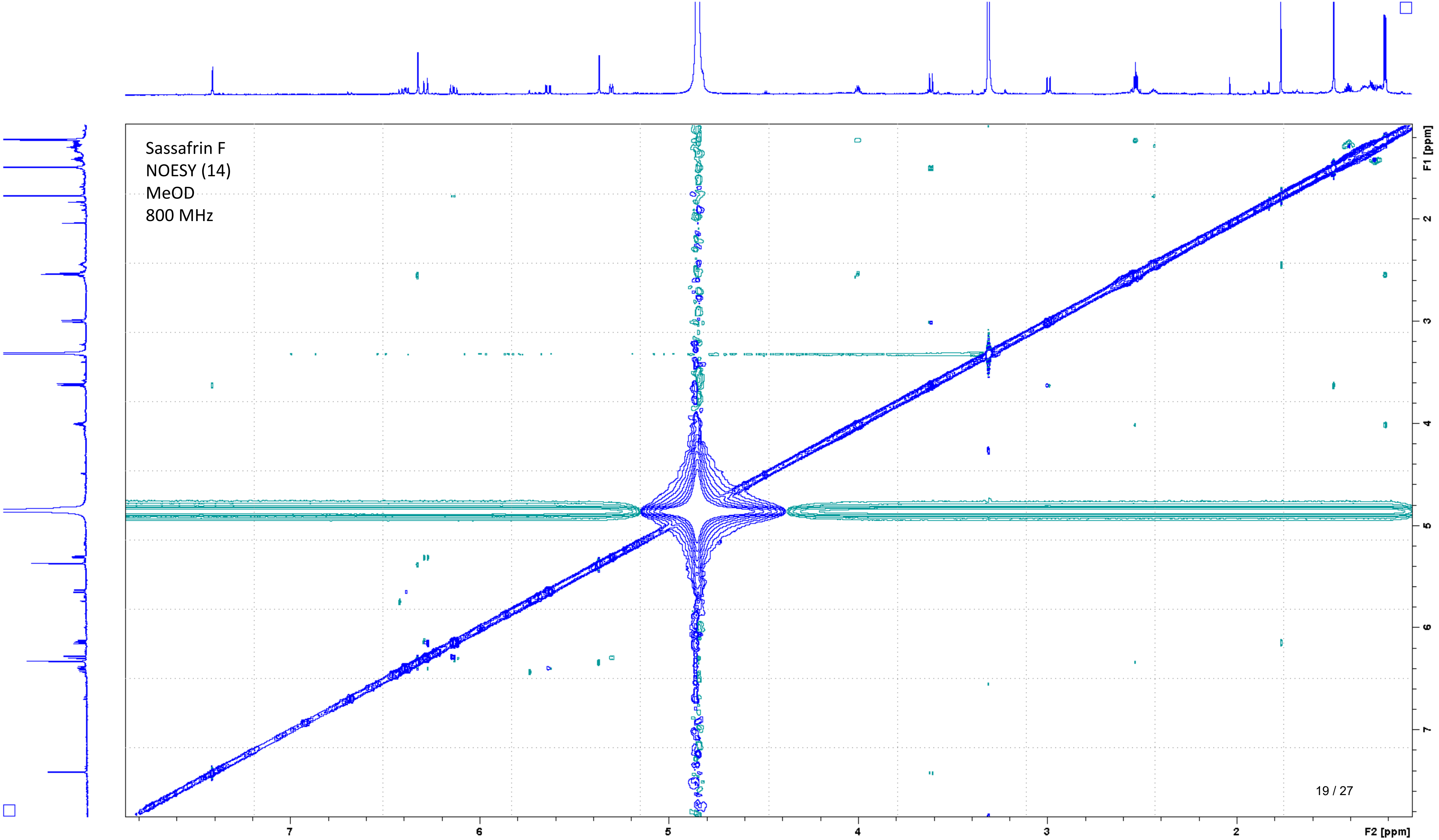

Sassafrin F
NOESY (14)
MeOD
800 MHz
F1 [ppm]
F2 [ppm]
2
3
4
5
6
7
7
6
5
4
3
2

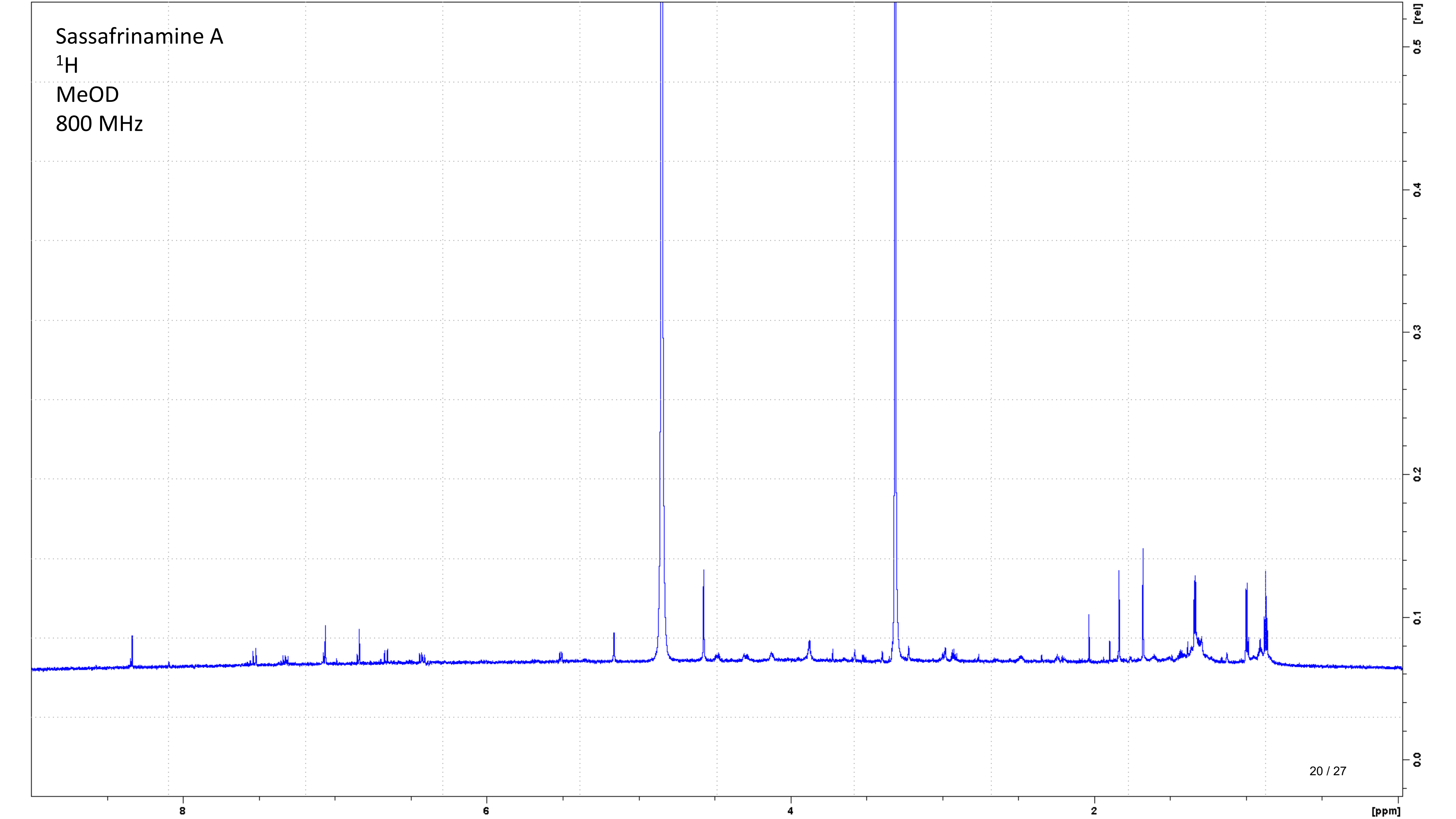
Sassafrinamine A
1H
MeOD
800 MHz
[rel]
0.5
0.4
0.3
0.2
0.1
0.0
8
6
4
2
[ppm]

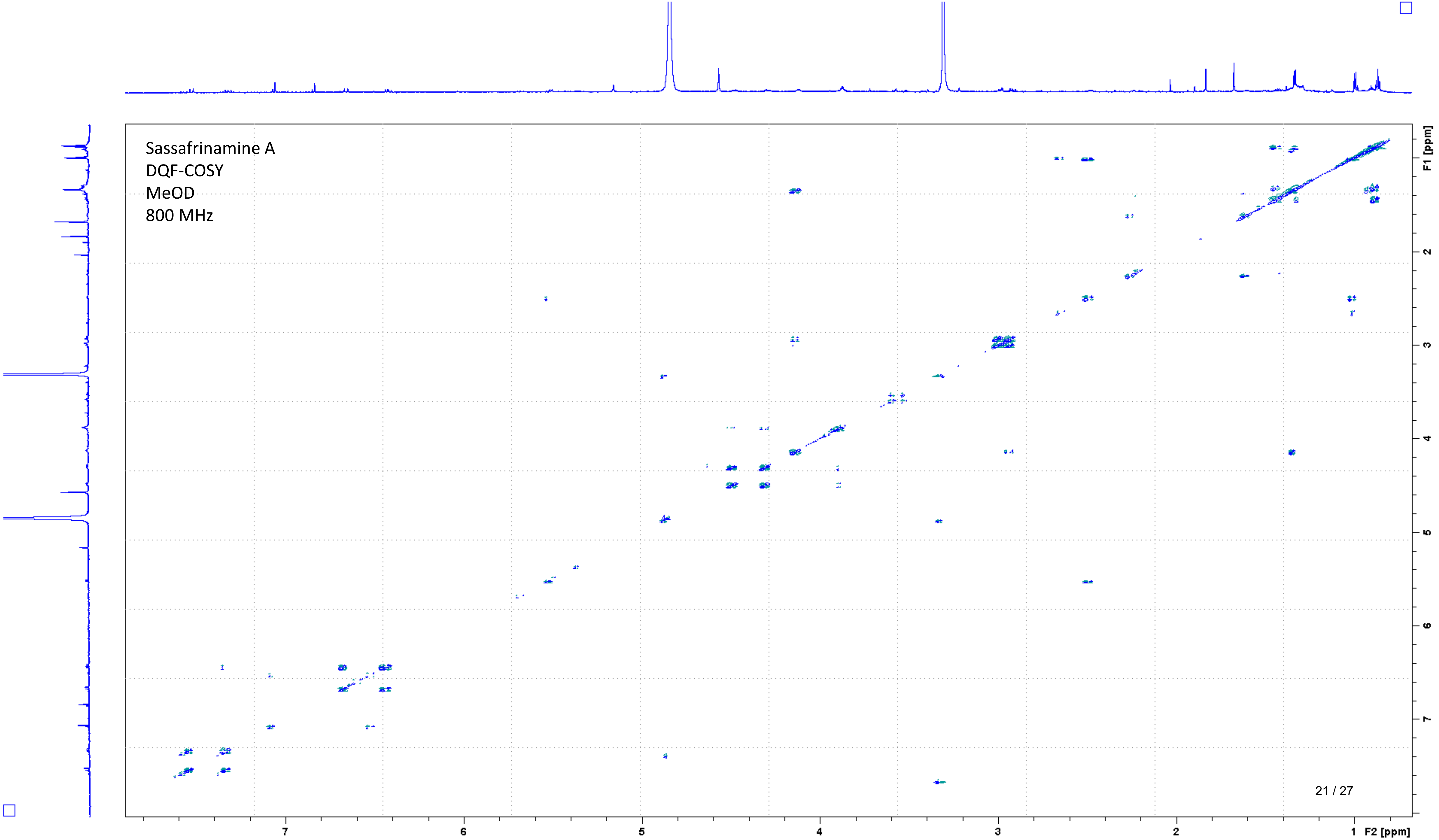

Sassafrinamine A
DQF-COSY
MeOD
800 MHz
F1 [ppm]
F2 [ppm]
7
6
5
4
3
2
1

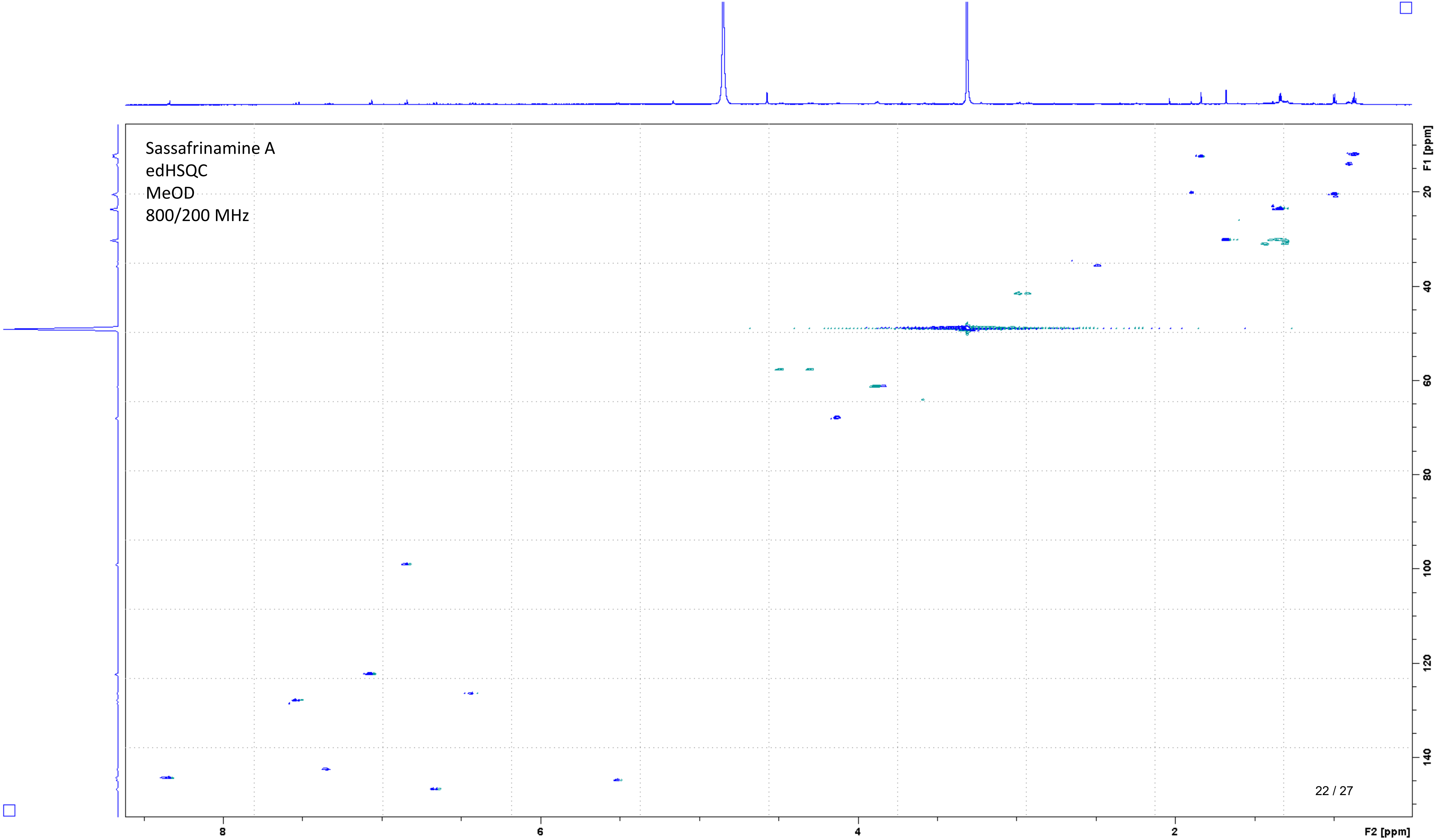
Sassafrinamine A
edHSQC
MeOD
800/200 MHz
F1 [ppm]
F2 [ppm]
20
40
60
80
100
120
140
8
6
4
2

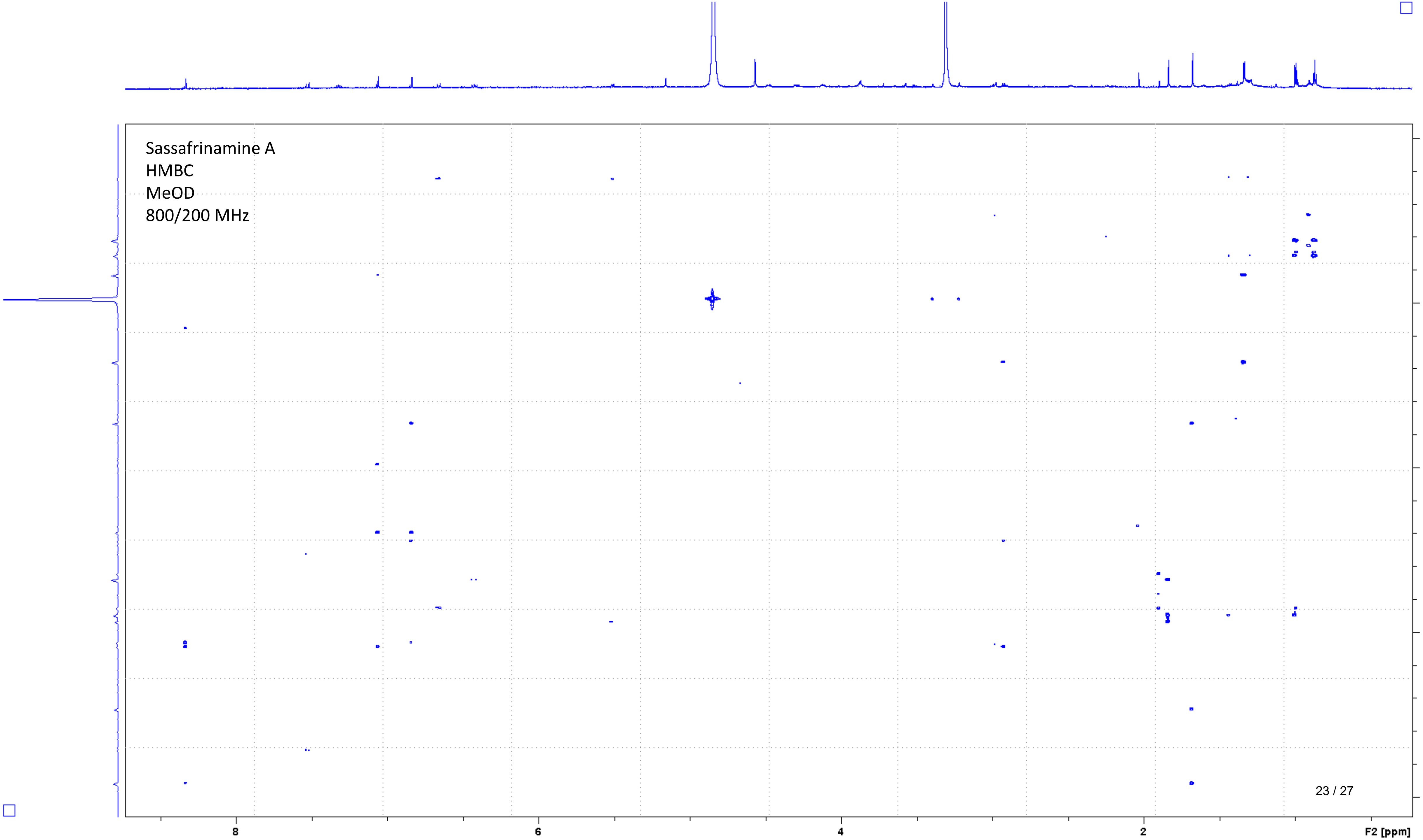
Sassafrinamine A
HMBC
MeOD
800/200 MHz
F1 [ppm]
50
100
150
200
8
6
4
2
F2 [ppm]

S3. Tandem mass spectra and proposed fragmentation pathway for deuterated sassafrin E.

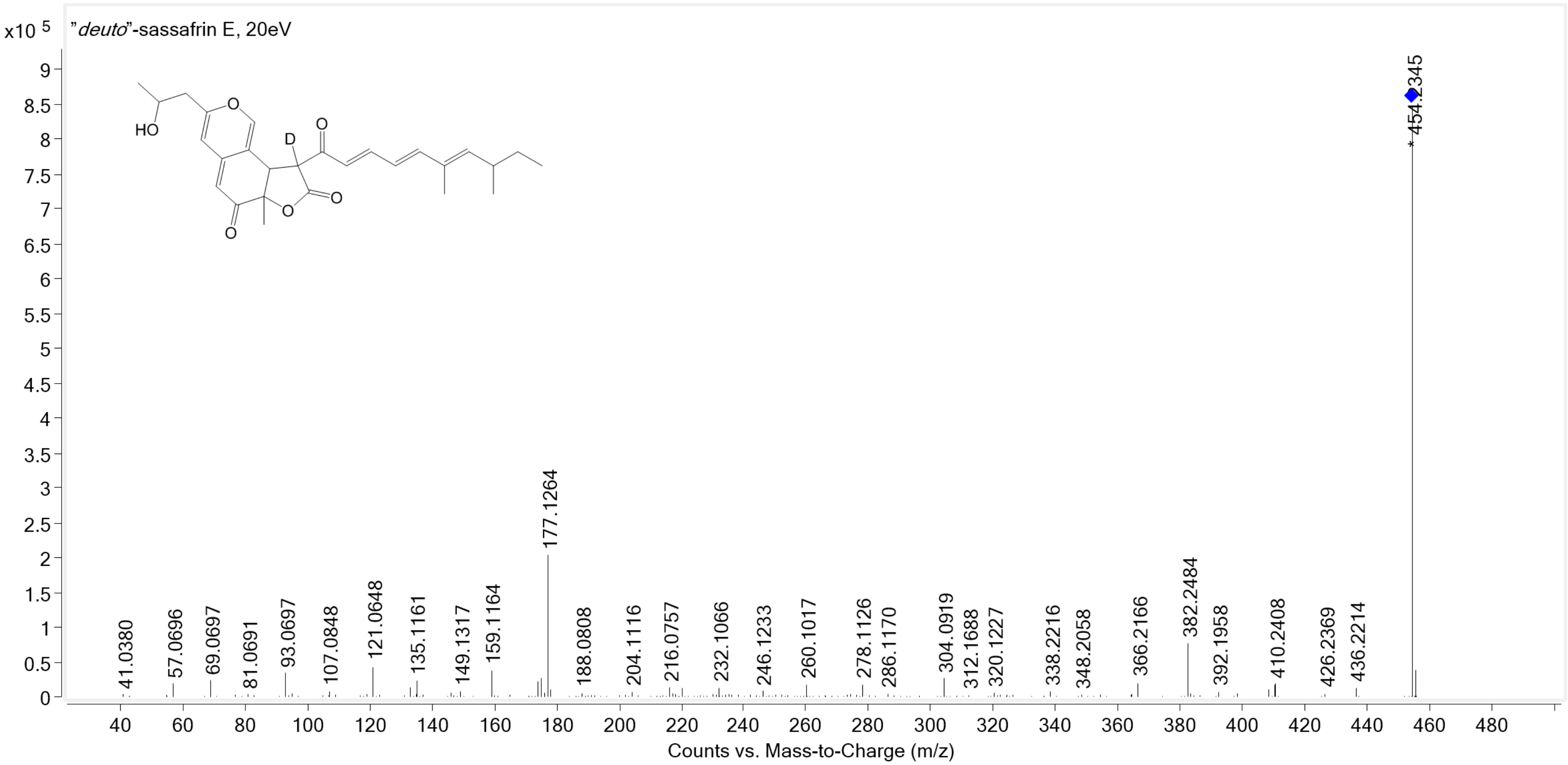

Figure S2. Tandem mass spectrum for deuterated sassafrin E at 20 eV colission energy.

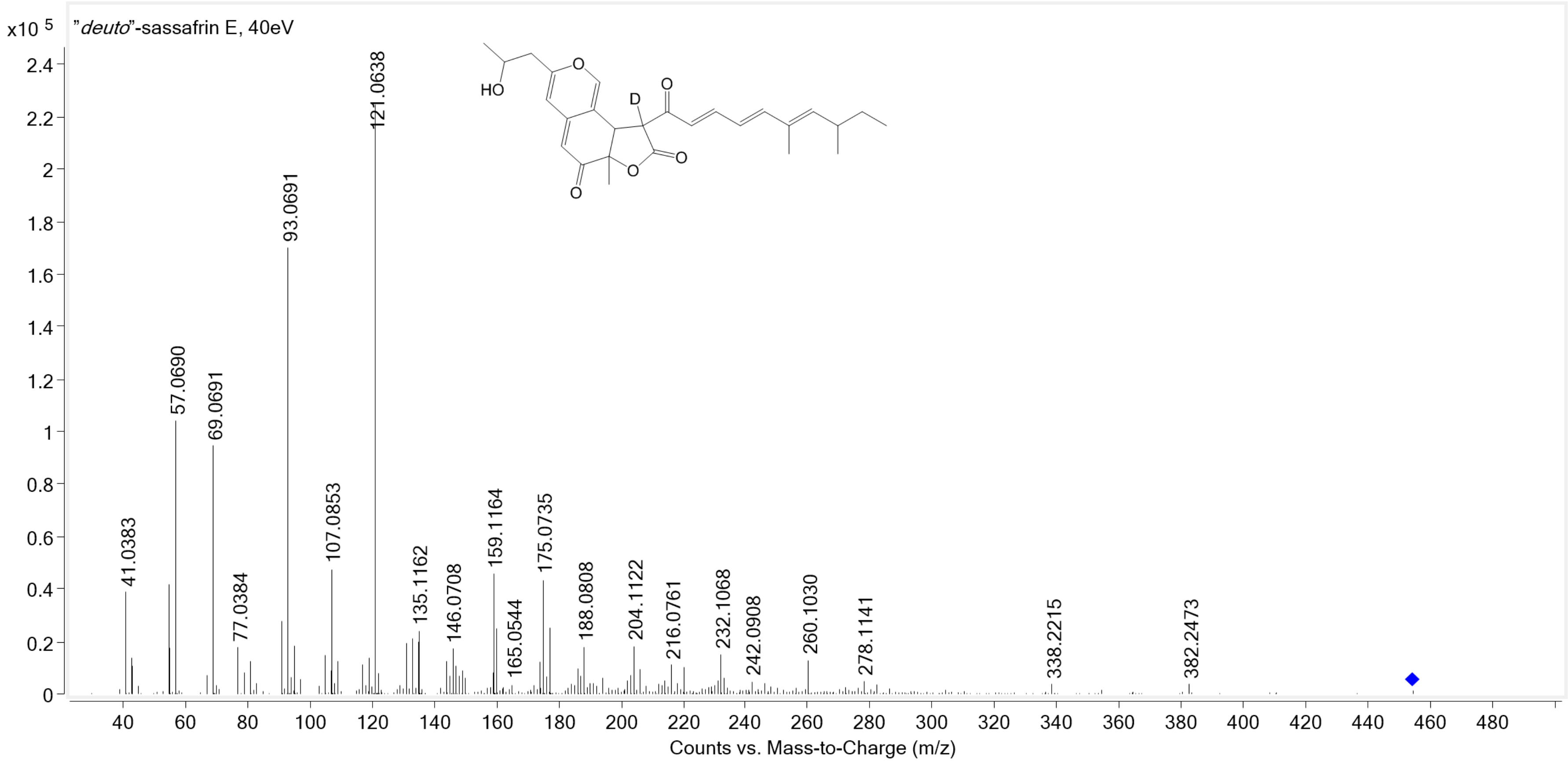

Figure S3. Tandem mass spectrum for deuterated sassafrin E at 40 eV colission energy

A)

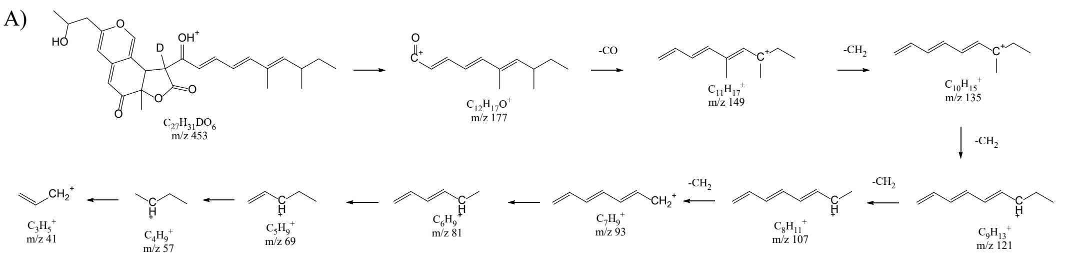

B)

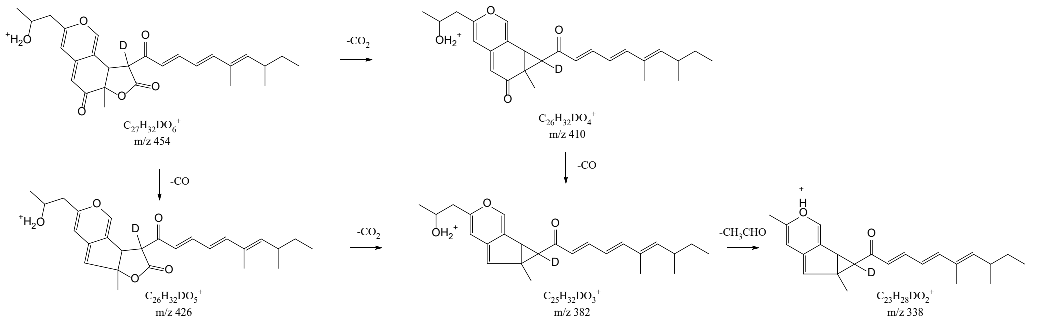

C)

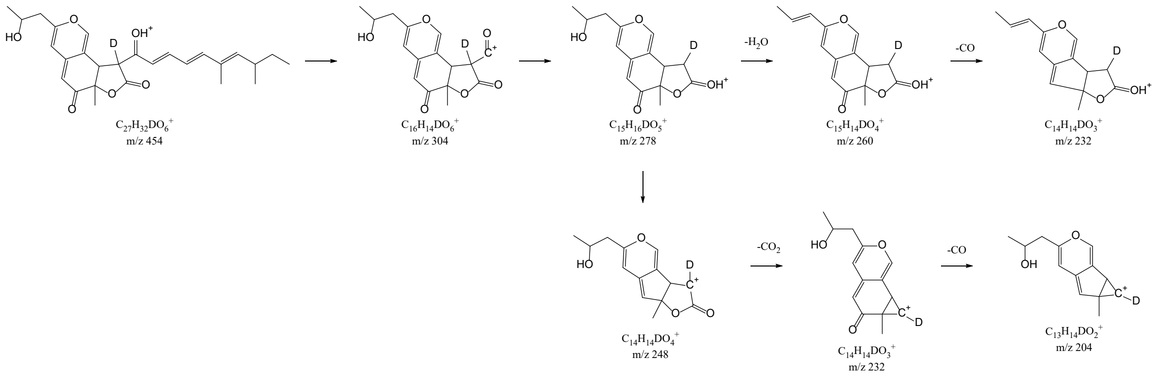

Figure S4. Proposed MS/MS fragmentation pathways for sassafrin E (1), based on deuteration in position H-23.

S4. Dereplication of mz453 compound in *Aspergillus neoglaber* compared to *Penicillium echinulatum*.

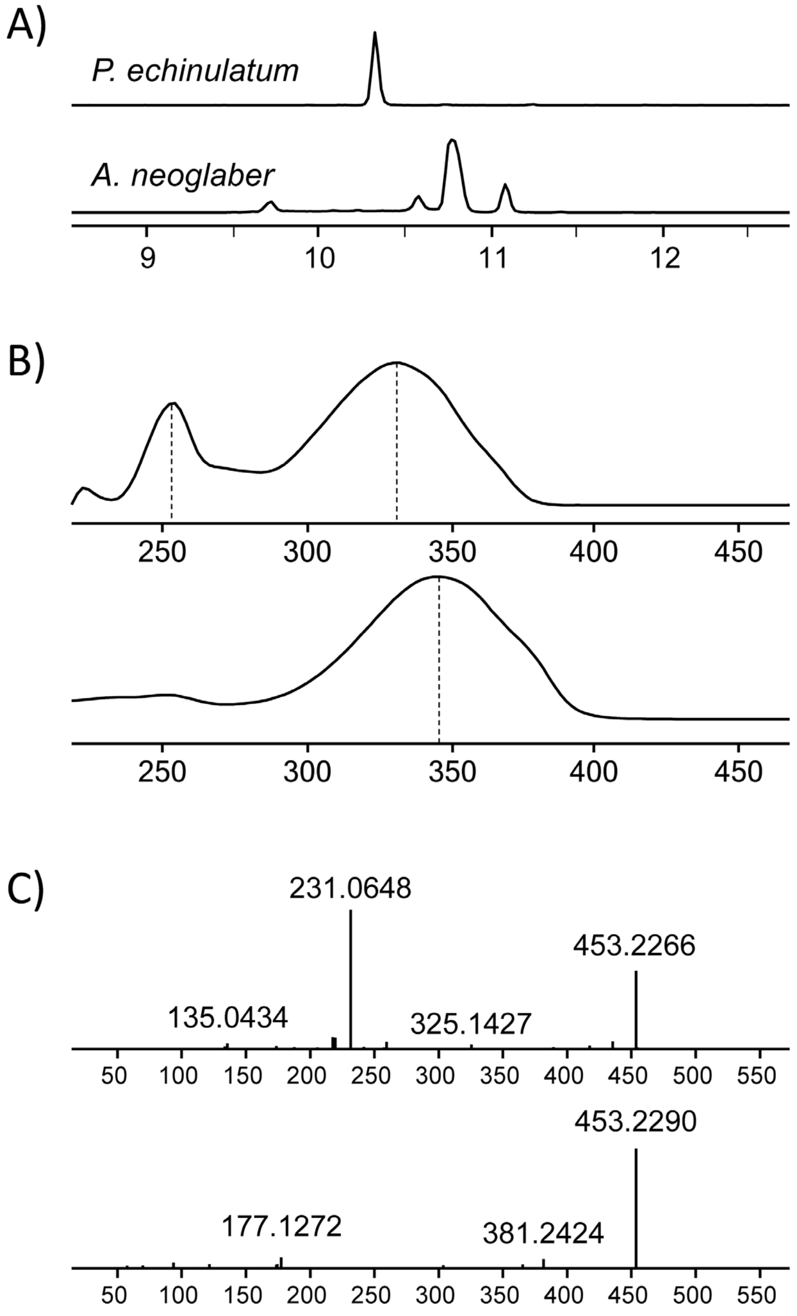

Figure S5. A) Extracted ion chromatogram (m/z 453.2277) for extracts from *P. echinulatum* and *A. neoglaber*. B) UV-VIS spectra for the mz453 compounds in *P. echinulatum* (top) and *A. neoglaber* (bottom). C) Tandem mass spectra (20 eV) for the mz453 compounds in *P. echinulatum* (top) and *A. neoglaber* (bottom).
